# Identification of 6ω-cyclohexyl-2-(phenylamino carbonylmethylthio)pyrimidin-4(3*H*)-ones targeting the ZIKV NS5 RNA dependent RNA polymerase

**DOI:** 10.3389/fchem.2022.1010547

**Published:** 2022-10-12

**Authors:** Guang-Feng Zhou, Cong-Qiang Xie, Jian-Xia Xue, Jing-Bo Wang, Yu-Zhuo Yang, Chang-Bo Zheng, Rong-Hua Luo, Ren-Hua Yang, Wen Chen, Liu-Meng Yang, Yue-Ping Wang, Hong-Bin Zhang, Yan-Ping He, Yong-Tang Zheng

**Affiliations:** ^1^ Key Laboratory of Bioactive Peptides of Yunnan Province/Key Laboratory of Animal Models and Human Disease Mechanisms of the Chinese Academy of Sciences, KIZ-CUHK Joint Laboratory of Bioresources and Molecular Research in Common Diseases, Kunming Institute of Zoology, Chinese Academy of Sciences, Kunming, Yunnan, China; ^2^ College of Pharmacy, Soochow University, Suzhou, China; ^3^ Key Laboratory of Medicinal Chemistry for Natural Resource, Yunnan Provincial Center for Research and Development of Natural Products, Ministry of Education, School of Pharmacy, Yunnan University, Kunming, China; ^4^ Medical College, Kunming University of Science and Technology, Kunming, Yunnan, China; ^5^ Yunnan Key Laboratory of Pharmacology for Natural Products, School of Pharmaceutical Science, Kunming Medical University, Kunming, China

**Keywords:** ZIKV, RdRp, acetylarylamine-S-DACOs, anti-ZIKV agent, NS5

## Abstract

Zika virus (ZIKV), a mosquito-borne flavivirus, is a global health concern because of its association with severe neurological disorders such as neonatal microcephaly and adult Guillain-Barre syndrome. Although many efforts have been made to combat ZIKV infection, there is currently no approved vaccines or antiviral drugs available and there is an urgent need to develop effective anti-ZIKV agents. In this study, 26 acetylarylamine-*S*-DACOs derivatives were prepared, and eight of them were found to have inhibitory activity against Zika virus. Among these substances, 2-[(4-cyclohexyl-5-ethyl-6-oxo-1,6-dihydropyrimidin-2-yl)thio]-N-(3,5-difluorophenyl)acetamide (**4w**) with the best anti-ZIKV activity was selected for in-depth study of antiviral activity and mechanism of action. Here, we discovered **4w** targeted on the ZIKV NS5 RNA -dependent RNA polymerase (RdRp), which exhibited good *in vitro* antiviral activity without cell species specificity, both at the protein level and at the RNA level can significantly inhibit ZIKV replication. Preliminary molecular docking studies showed that **4w** preferentially binds to the palm region of NS5A RdRp through hydrogen bonding with residues such as LYS468, PHE466, GLU465, and GLY467. ZIKV NS5 RdRp enzyme activity experiment showed that **4w** could directly inhibit ZIKV RdRp activity with EC_50_ = 11.38 ± 0.51 μM. In antiviral activity studies, **4w** was found to inhibit ZIKV RNA replication with EC_50_ = 6.87 ± 1.21 μM. ZIKV-induced plaque formation was inhibited with EC_50_ = 7.65 ± 0.31 μM. In conclusion, our study disclosed that acetylarylamine-*S*-DACOs is a new active scaffolds against ZIKV, among which compound **4w** was proved to be a potent novel anti-ZIKV compound target ZIKV RdRp protein. These promising results provide a future prospective for the development of ZIKV RdRp inhibitors.

## 1 Introduction

Zika virus (ZIKV) is an important member of genus Flavivirus which includes dengue virus (DENV), West Nile virus, yellow fever virus (YFV), hepatitis C virus (HCV) and Japanese encephalitis virus (Dick G et al., 1952; Barrows et al., 2018). ZIKV is transmitted primarily by mosquito bites, however vertical, sexual, transfusion, and transplantation transmissions have been also related (Li et al., 2012; Gregory et al., 2017). Since the worldwide outbreak of ZIKV in 2015, it has caused a serious threat to human health due to its clinical symptoms that are different from other flaviviruses, such as neonatal microcephaly, adult Guillain-Barre syndrome, and other serious neurological diseases (Petersen et al., 2016; Cumberworth et al., 2017; Bhagat et al., 2021). ZIKV is not a new type of virus, and its spread in human populations has a long history. However, the research on vaccines and antiviral drugs in recent years has not really prevented its spread, but the development of some vaccines and drugs has always stayed in the stage of drug clinical research. At present, there are no reliable vaccines and drugs that can be clearly used for the treatment and prevention of ZIKV infection (Boldescu et al., 2017; Kraemer et al., 2019). Therefore, the development of effective antiviral drugs is one of the most important strategies to inhibit ZIKV infection.

ZIKV is a single-stranded positive-stranded RNA virus with spherical virions, with a diameter of about 40 nm and an envelope (Guo et al., 2021). The viral core consists of viral genomic RNA and capsid proteins, and its surface proteins are arranged in icosahedral symmetry (Hu and Sun, 2019). The ZIKV genome is about 11 kb in length, including two flanking noncoding regions and one open reading frame. The ZIKV genome consists of three structural proteins (capsid protein, envelope protein, and prM protein) and seven non-structural proteins (NS1, NS2A, NS2B, NS3, NS4A, NS4B, and NS5) (Sirohi and Kuhn, 2017). NS5 is essential for the replication of theZIKV RNA genome. The N-terminal portion of NS5 contains a methyltransferase (MTase), followed by a short linker that connects to the RNA-dependent RNA polymerase (RdRp), an enzyme that plays an important role in viral RNA replication, acting metalloproteinases (Lim et al., 2013). Regarding the function of the ZIKV RdRp subdomain, it was found that the palm subdomain mainly acts as a catalytic center (Züst et al., 2013) and mediates RNA template binding, translocation, and nucleoside triphosphate (NTP) specificity, as well as nucleotide transfer and priming Alignment of Nucleotide ATP (Selisko et al., 2012; Duan et al., 2017; Shu and Gong, 2017). In addition, the thumb domain has an initiation loop, which is primarily responsible for facilitating the initiation of ATP-specific RNAs and regulating the transition of subsequent elongation steps (Bartholomeusz and Thompson, 1999). The finger domain is involved in the formation of the active site and NTP entry channel by controlling the *de novo* synthesis of RdRp (Akiyama et al., 2021; Li and Kang, 2022). Finally, alanine substitutions have demonstrated that several N-pocket residues are critical for NS5 polymerase *de novo* activity as well as viral replication (Ackermann and Padmanabhan, 2001; Malet et al., 2008). Due to its pivotal role in viral replication, RdRp is a promising drug target for ZIKV infection (Malet et al., 2008; Lim et al., 2015; Ferrero et al., 2019).

**FIGURE 1:**
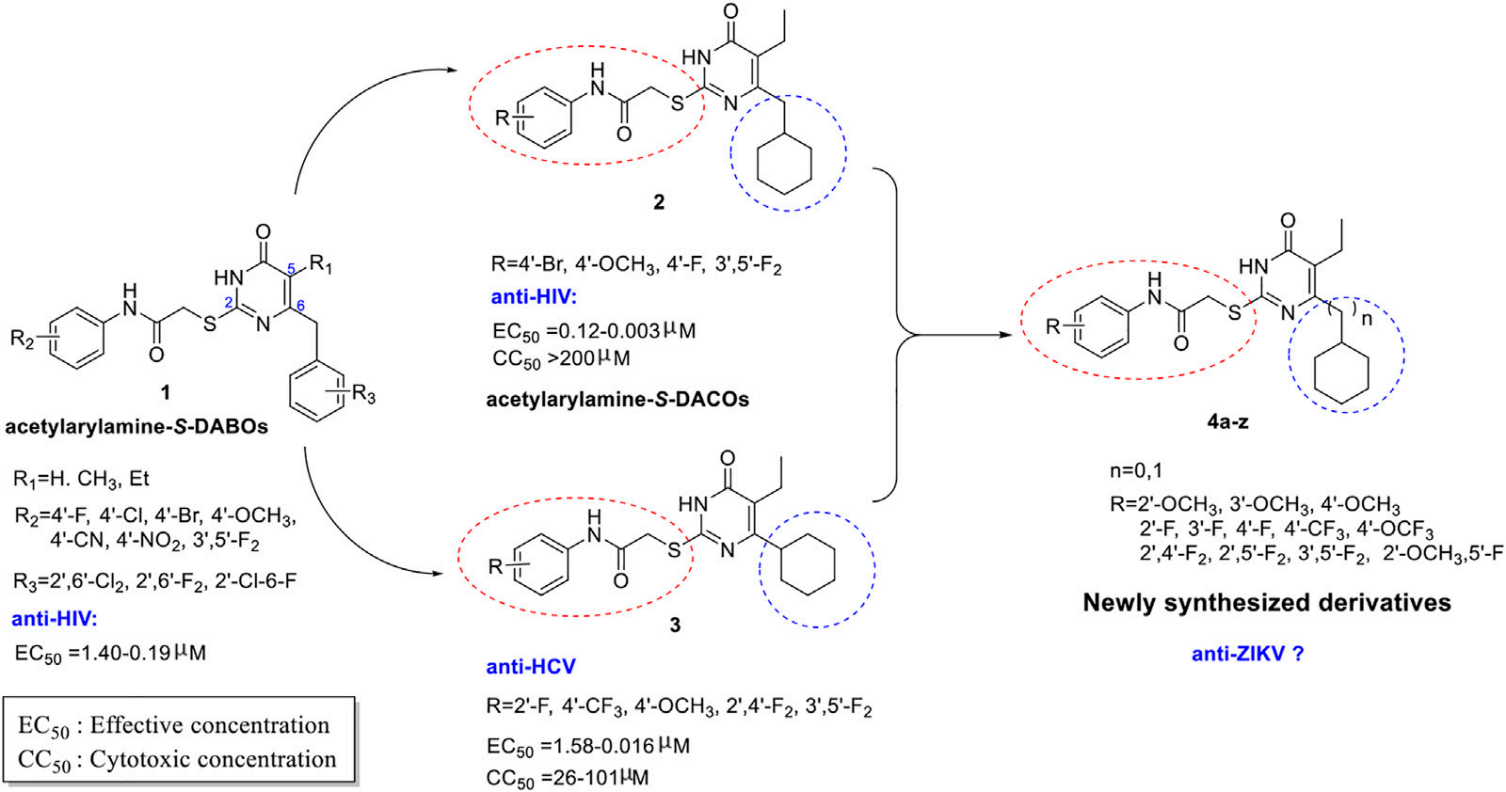
The chemical structure of the acetylarylamine-*S*-DABOs compounds.

5-Alkyl-6-aryl-2-(phenylaminocarbonylmethylthio)pyrimidin-4(3*H*)-ones (acetylarylamine-*S*-DABOs general structure **1**, Figure 1) are a class of excellent non-nucleoside reverse transcriptase inhibitors (NNRTIs) with high broad-spectrum HIV-1 inhibitory activity (Yu et al., 2009; Yu et al., 2011). In our previous antiviral studies, starting from compounds of general structure **1**, we replaced the aromatic ring at the *C*-6 position of the pyrimidine by a cyclohexyl moiety, obtaining a series of 5-Alkyl-6-cyclohex-yl-2-(phenylaminocarbonylmethylthio)pyrimidin-4(3*H*)-ones (acetylarylamine-*S*- DACOs general structure **2**, Figure 1) which can inhibit HIV replication in the low nanomolar range (He et al., 2011), while compounds of general structure **3** showed good anti-HCV activity (He et al., 2015). In recent years, we have launched our search of new active scaffolds against flaviviruses such as HCV, DENV and ZIKV (Wu et al., 2017; Qian et al., 2022; Rui et al., 2022). Given the success of drug development against HCV infection and the presence of HCV homologs with ZIKV, the repurposing of HCV inhibitors for ZIKV is an attractive starting point for the discovery of a novel skeleton of anti-ZIKV drugs. By comparing the structures of the **2** and **3** series compounds, it can be seen that the compounds with F or OCH_3_ substituent on the benzene ring at the end of *C*-2 side chain have good anti-HIV or HCV activity. Therefore, in the design of target molecules, fluoro-containing substituents such as F, CF_3_, OCF_3_ and OCH_3_ were selected and introduced into different positions on the benzene ring. In addition, the *C*-6 substituent also had a significant effect on the antiviral activity of the compounds. For example, *C*-6 cyclohexylmethyl substituted compounds **2** have anti-HIV activity, while *C*-6 cyclohexyl substituted compounds **3** have anti-HCV activity. Therefore, we intend to synthesize *C*-6 cyclohexyl or *C*-6 cyclohexylmethyl substituted acetylaryl-amine-*S*-DACOs target molecules simultaneously. Therefore, using compounds of general structures **2** and **3** as lead compounds, a series of new acetylarylamine-*S*-DACO derivatives **4a-z** (Figure 1) were designed by introducing selective substituents (R) on the ω-phenyl group of the pyrimidine ring *C*-2 side chain and a cyclohexyl or cyclohexyl methyl at the *C*-6 position. In this paper, we describe the synthesis, cell-based and target-based activity evaluation of the series of acetylarylamine-*S*-DACOs derivatives against ZIKV infection.

## 2 Results and discussion

### 2.1 Chemistry

**SCHEME 1:**
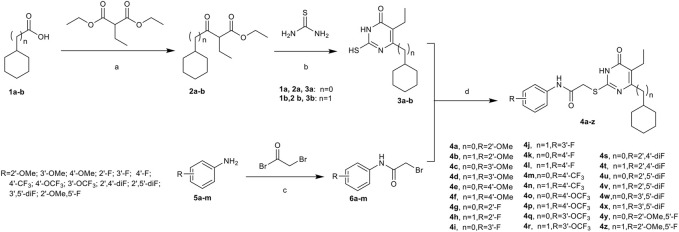
Synthesis of Compounds **4a-z**. Reagents and conditions:**(A)** (1)KOH, EtOH, r.t., overnight; (2) CDI, MgCl_2_, Et_3_N, CH_3_CN, r.t., 1 h, then refluxed for 8–12 h; **(B)** EtONa, dry EtOH, reflux, 6–12 h; **(C)** Et_3_N, CH_2_Cl_2_; **(D)** K_2_CO_3_, DMF, r.t., 6–10 h.

A series of acetylarylamine-*S*-DACOs **4a-z** was synthesized as illustrated in Scheme 1. Following the procedure described previously (He et al., 2004; He et al., 2011; Wu et al., 2020; Li et al., 2021), the key intermediates *β*-Ketoesters **2a/b** were prepared by exposure of each commercially available cyclohexanecarboxylic acid **1a** or 2-cyclohexylacetic acid **1b** to 1,1′-carbonyl-diimidazole (CDI) followed by treatment with ethyl potassium malonates in the presence of anhydrous MgCl_2_ and Et_3_N. Subsequent condensation of **2a/b** with thiourea in the presence of EtONa in refluxing EtOH afforded 5,6-disubstituted thiouracils **3a/b**. Next by selective *S-*alkylation of **3a/b** with the appropriate N-phenylacetamide halides **6a-m** in the presence of anhydrous K_2_CO_3_ in anhydrous DMF afforded the desired target compounds **4a-z**. These compounds were characterized by ^1^H NMR and HRMS for their structural accuracy.

### 2.2 Anti-ZIKV activity evaluation

Initially, the novel acetylarylamine-*S*-DACOs **4a-z** were tested for their cytotoxicity and anti-ZIKV activity in Vero cells by plaque formation according to previously detailed procedure (Xu et al., 2017). The activity data was interpreted in CC_50_ values (cytotoxicity), EC_50_ (anti-ZIKV activity) and SI (selectivity index, given by the CC_50_/EC_50_ ratio) (Table 1). As show in Table 1, the preliminary plaque results revealed that there are eight active compounds against ZIKV replication with EC_50_ < 20 μM and CC_50_ > 90 μM, ensuring that antiviral effect of these compounds is not related to their toxicity. The most potent compounds were **4j**, **4n**, and **4w** with EC_50_ values of 7.48, 7.75, and 7.65 μM, respectively. They were about 6 times more potent than the reference drug Ribavirin (EC_50_ = 48.88 μM). From Table 1, we can make a preliminary summary of this series of compounds: First, the type of substituent R on benzene ring has a significant impact on the anti-ZIKV activity of these compounds. For example, the R of the above eight compounds showing anti-ZIKV were all fluorine substituents, while the compounds **4a-f**, **4y**, and **4z** obtained by introducing methoxyl onto benzene ring did not show activity. In addition, the position of the substituent R on the benzene ring also has an important influence on the activity. For example, the activity of the 3′-F-substituted compound **4j** (EC_50_ = 7.48 μM) is better than that of the 2′-F-substituted analog **4h** (EC_50_ = 10.77 μM), while the 4′-F-substituted analog **4l** becomes inactive compound. The 3′-OCF_3_-substituted compound **4r** can inhibit ZIKV with EC_50_ value of 8.84 μM, while its 4′-OCF_3_-substituted counterpart **4p** is inactive against ZIKV. At last, the length (n) of the connecting carbon chain between the *C-*6 site of the pyrimidine ring and cyclohexane also affects the activity of the compound, and there may be a synergistic relationship between n and substituent R. For example, compound **4j** (R = 3′-F, *n* = 1), **4n** (R = 4′-CF_3_, *n* = 1), and **4r** (R = 3′-OCF_3_, *n* = 1) are more active than their counterpart **4i** (R = 3′-F, *n* = 0), **4m** (R = 4′-CF_3_, *n* = 0), and **4q** (R = 3′-OCF_3_, *n* = 0), respectively, showing cyclohexylmethyl (*n* = 1) is more favorable for activity. Unlike SAR of 3′-F or 3′-OCF_3_ series, **4x** (R = 3′,5′-diF, *n* = 1) is less active than **4w** (R = 3′,5′-diF, *n* = 0), showing cyclohexyl (*n* = 0) is more favorable for activity.

**TABLE 1:** Structures, EC_50_ ^a^, CC_50_ ^b^, and SI of titled compounds.

Compd.	R	n	EC_50_ (μM)	CC_50_ (μM)	SI
**4a**	2′-OMe	0	NA^c^	>200^d^	/^e^
**4b**	2′-OMe	1	NA	>200	/
**4c**	3′-OMe	0	NA	109.32 ± 3.19	/
**4d**	3′-OMe	1	NA	130.66 ± 5.49	/
**4e**	4′-OMe	0	NA	>200	/
**4f**	4′-OMe	1	NA	>200	/
**4g**	2′-F	0	16.29 ± 1.31	>200	>12.27
**4h**	2′-F	1	10.77 ± 1.67	136.8 ± 2.41	12.70
**4i**	3′-F	0	19.37 ± 0.38	>200	>10.33
**4j**	3′-F	1	7.48 ± 0.67	146.9 ± 1.01	19.64
**4k**	4′-F	0	NA	>200	/
**4l**	4′-F	1	NA	122.5 ± 5.19	/
**4m**	4′-CF_3_	0	NA	>200	/
**4n**	4′-CF_3_	1	7.75 ± 1.35	89.98 ± 1.35	11.62
**4o**	4′-OCF_3_	0	NA	>200	/
**4p**	4′-OCF_3_	1	NA	163.5 ± 7.34	/
**4q**	3′-OCF_3_	0	NA	>200	/
**4r**	3′-OCF_3_	1	8.84 ± 0.72	165.5 ± 2.38	18.73
**4s**	2′,4′-diF	0	NA	>200	/
**4t**	2′,4′-diF	1	10.51 ± 1.70	163.0 ± 5.76	15.51
**4u**	2′,5′-diF	0	NA	>200	/
**4v**	2′,5′-diF	1	NA	100.9 ± 5.03	/
**4w**	3′,5′-diF	0	7.65 ± 0.31	>200	>26
**4x**	3′,5′-diF	1	NA	60.25 ± 2.02	/
**4y**	2′-OMe,5′-F	0	NA	150.0 ± 0.86	/
**4z**	2′-OMe,5′-F	1	NA	>200	/
Ribavirin			48.88 ± 3.42	>200	>4.09

^a^ Plaque assay result conducted independently in triplicate.

^b^ MTT result conducted independently in triplicate.

^c^ “NA” represents “not active by the plaque assay that 50 μM of testing compound expresses less than 50% inhibition of ZIKV replication.”

^d^ Indicated concentration is the highest soluble concentration which does not reach 50% cytotoxicity.

^e^ “/” means “not available.”

**FIGURE 2:**
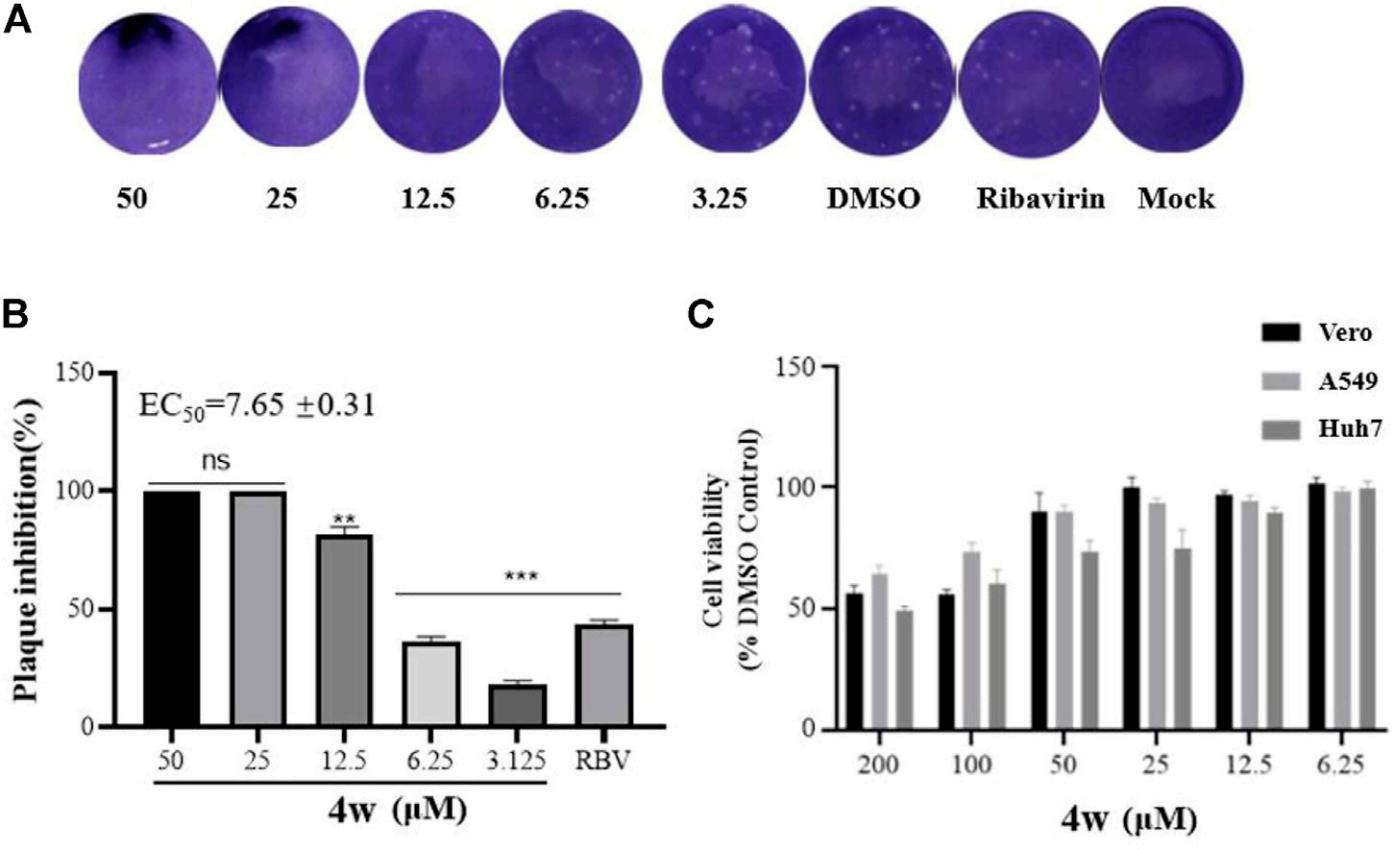
Compounds **4w** inhibited the plaque formation caused by ZIKV infection, Vero cells were infected with ZIKV (MOI = 0.1) at 37°C for 2 h. **(A)** Original graph of **4w** inhibiting ZIKV plaque formation; **(B) 4w** inhibition rate of ZIKV-induced plaque formation; **(C) 4w** cytotoxicity to Vero, Huh7, and A549 cells, data is the mean (±SD) of three experiments, with DMSO as a positive control, and Ribavirin = 50 μM as a positive drug control. *p < 0.05; **p < 0.01; ***p < 0.001.

The strongest plaque inhibition by compound **4w** is shown in Figure 2. As we can see from Figure 2A, **4w** significantly inhibited the plaque formation of ZIKV, and this inhibitory effect was positively correlated with the concentration of **4w**. It inhibited ZIKV plaque formation with EC_50_ = 7.65 ± 0.31 μM (Figure 2B). We tested its cytotoxicity to A549, Huh7 and Vero cells by MTT method, and found that it had no obvious toxicity to these three different cell lines, the CC_50_ was greater than 200 μM (Figure 2C), and its SI value was greater than 26, which suggested that **4w** could be used as a new type of anti-ZIKV active compound for further development and research. Subsequently, compound **4w**, as a novel anti-ZIKV skeleton, was subjected to further analysis to detect the antiviral effects and elucidate the drug target and the mode of drug action.

### 2.3 Compound 4w interacts with ZIKV RdRp directly

**FIGURE 3:**
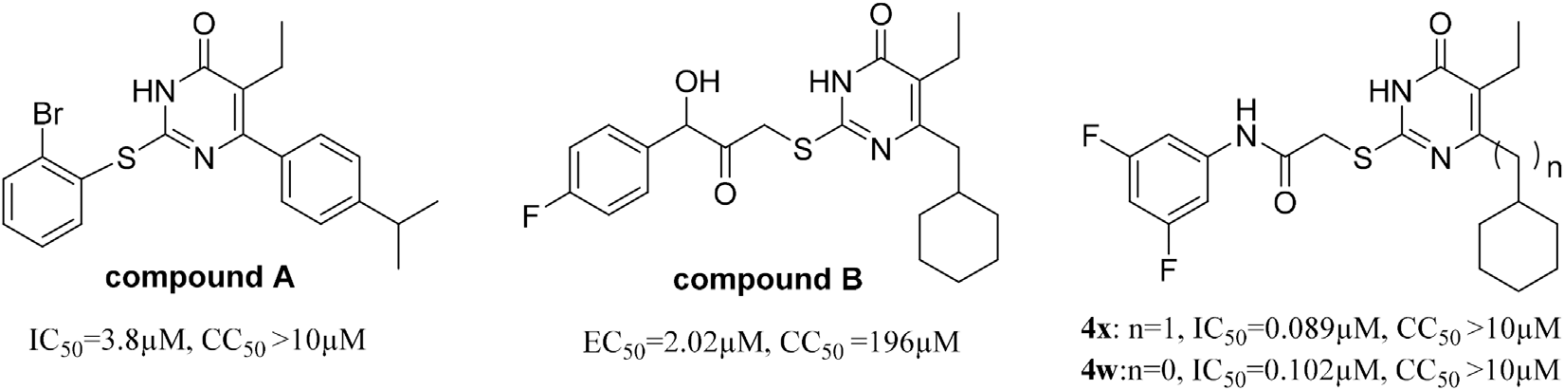
Pyrimidones analogues active against HCV targeting NS5B RdRp.

**FIGURE 4:**
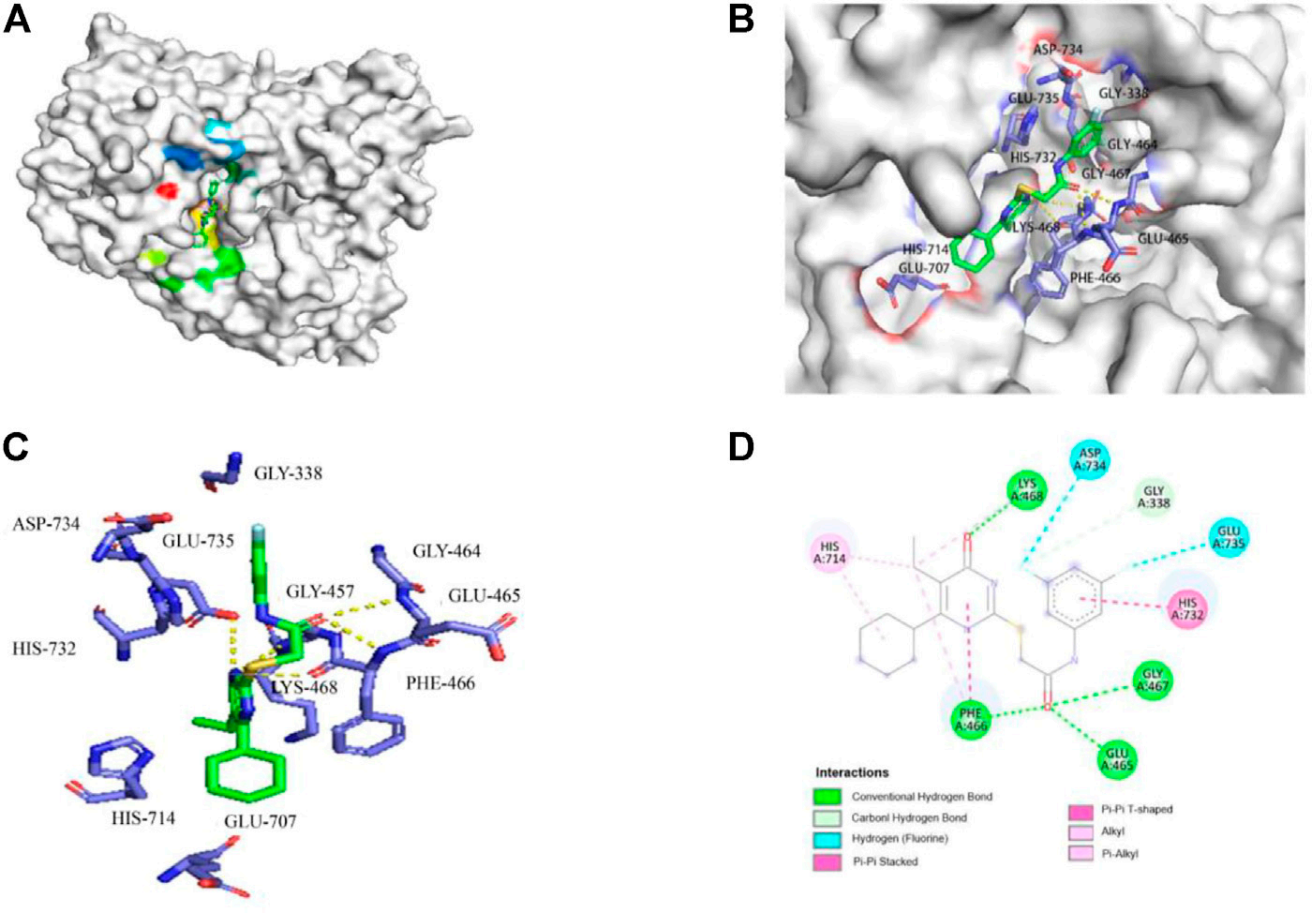
Molecular docking model of **4w** and ZIKV RdRp. **(A) 4w** binds to ZIKV RdRp with lower free energy, the compound is shown in stick form, and the amino acid interaction site is shown as surface structure; **(B) 4w** binds to ZIKV RdRp, the compound is shown in green, interacts amino acids are shown in purple; **(C)** The three-dimensional structure of the interaction between **4w** and ZIKV RdRp is magnified, **4w** is shown in green, the interacting amino acids are shown in purple, and the intermolecular hydrogen bond force is marked with a yellow dotted line; **(D)** The interaction map between **4w** and each amino acid of ZIKV RdRp protein, the intermolecular hydrogen bond force is shown in green.

Among the viral targets, NS5 polymerase is one of the most promising and exploited targets, being highly conserved among flaviviruses. Previously, some related pyrimidones have been identified as potential HCV inhibitors targeting NS5 RdRp. For example, Ding et al. have reported 5-cyano-6-aryl-2-thiouracil (compound A, Figure 3) as a potent inhibitor of HCV NS5B RdRp (IC50 = 3.8 μM) (Ding et al., 2006). Wu et al. (2017) identified 2-hydroxylphenethyl sulfanyl-oxopyrimidines derivatives as potential anti-HCV agent targeting HCV NS5B RdRp (e.g., compound B, Figure 3). After our research group revealed the potent anti-HCV activity of acetylarylamine-*S*-DACO derivatives (general structure **3**, Figure 1), Shen (2015) further determined that this series of compounds targeted HCV NS5B RdRp. For example, compound **4x** and **4w** (Figure 3) as a potent inhibitor of HCV NS5B RdRp have IC_50_ of 0.089 μΜ and 0.102 μM. Since compound **4w** has already been shown to be a HCV RdRp inhibitor, we speculated compound **4w** may also act on ZIKV RdRp.We first detected whether **4w** could interact with ZIKV RdRp by molecular docking method, and found that **4w** can bind to ZIKV RdRp domain with low free energy (Figures 4A,B). ZIKV RdRp is mainly composed of three domains, namely palm domain (amino acid regions 321–488 and 542–608), finger domain (amino acid regions 489–541 and 609–714) and thumb (amino acid regions 715–903) (Chen et al., 2021). In the docking results, it was found that **4w** mainly binds to the palm domain of ZIKV RdRp, and preferentially binds to LYS468, PHE466, GLU465, and GLY467 amino acids in the palm region by hydrogen bonding force (Figures 4C,D). Details are as follows: 1) The pyrimidine ring of compound **4w** is immobilized in the binding cavity by hydrogen bonding between its *C*-4 carbonyl group and LYS468 and π-π interaction with the benzene ring of PHE466. 2) The *C-2β*-Carbonyl of **4w** formed triple hydrogen bonds with GLU465, PHE466 and GLY467, thus facilitating stable binding of the inhibitor to ZIKV RdRp;3) The *C*-2 terminal 3, 5-2F-phenyl group of **4w** points to the region surrounded by the side chains of HIS732, GLU735, and ASP734. In addition to the π-π interaction between the benzene ring and the imidazole ring of HIS732, the two F atoms on the benzene ring also form the F-H interaction with the two polarized CH groups on GLU735 and ASP734, respectively, which further strengthens the combination of **4w** and ZIKV RdRp.

### 2.4 Compound 4w interacts with ZIKV NS5 protein

Cell thermal shift assay (CETSA) experiments are often used to detect the binding of intracellular drugs to target proteins. The assay was based on the principle of ligand-induced changes in protein thermal stability. The stability of the protein is evaluated by comparing the melting curves between the control group and the experimental group, thus assessing the interaction of the compound with the target protein.

**FIGURE 5:**
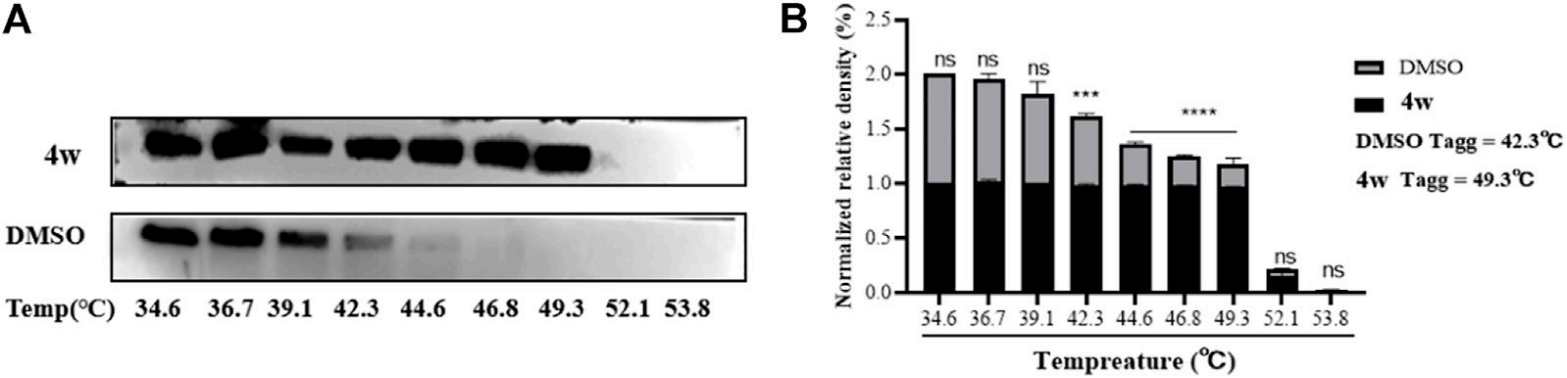
The interaction between **4w** and ZIKV NS5 protein. **(A)** WB detection of ZIKV NS5 protein expression under different temperature gradients after **4w** treatment; **(B)** NS5 protein grayscale analysis and ZIKV NS5 protein expression after **4w** treatment observed changes in aggregation temperature; data is the mean (±SD) of three experiments. *p < 0.05; **p < 0.01; ***p < 0.001; ****p < 0.0001.

Molecular docking experiments, suggested that **4w** interacts with ZIKV RdRp domain, and ZIKV RdRp domain is the *C*-terminal subdomain of ZIKV NS5 protein. Therefore, in order to further verify the interaction between **4w** and ZIKV NS5, ZIKV-infected cell were treated with **4w** (at 50 μM concentration), then total protein was incubation for 60 min at room temperature, and it was divided into nine equal parts. The same volume of DMSO was added as a control, and the treated protein was heated in a temperature gradient and then detected ZIKV NS5 protein by WB assay (Figure 5A). It was found that **4w** can bind to ZIKV NS5 protein in cells, thereby improving the thermal stability of ZIKV NS5 protein and preventing its aggregation and precipitation at high temperature. The apparent aggregation temperature (Tagg) of ZIKV NS5 protein was increased from 42.3°C to 49.3°C (Figure 5B).

### 2.5 Compound 4w inhibits ZIKV RdRp activity

**FIGURE 6:**
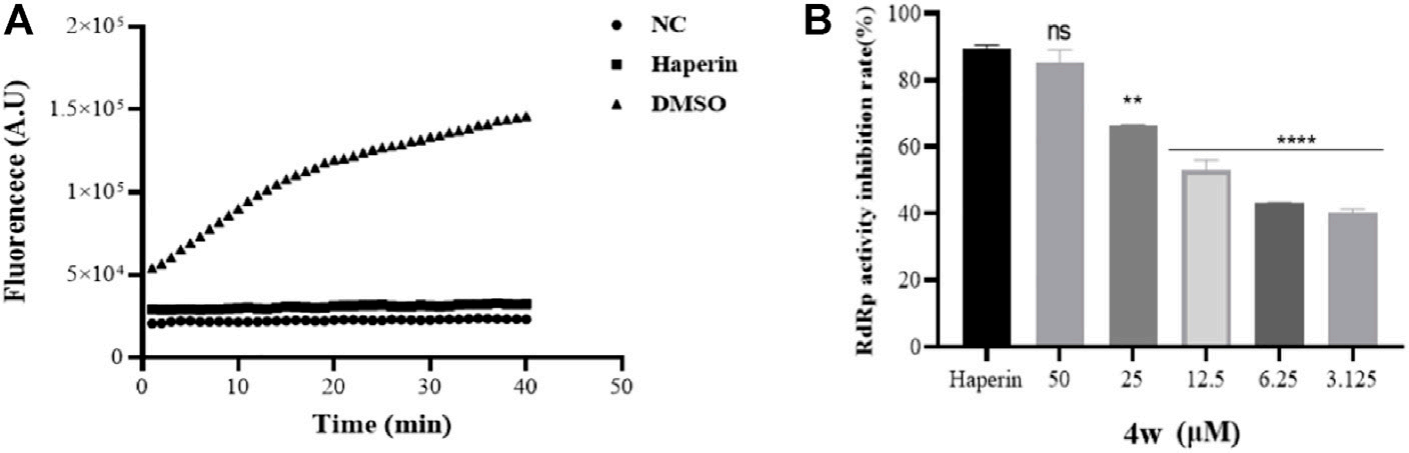
Compound **4w** inhibits ZIKV RdRp activity. **(A)** ZIKV RdRp activity assay, NC is the negative control with substrate only; DMSO is the compound solvent control, Haperin is the positive drug control; **(B) 4w** concentration gradient Inhibition of ZIKV RdRp protein. Data is the mean (±SD) of three experiments, with DMSO as a positive control, and Heparin = 5 μM as a positive drug control. *p < 0.05; **p < 0.01; ***p < 0.001; ****p < 0.0001.

Through molecular docking experiments and cell thermal shift analysis, we supose that **4w** may have a direct interaction with ZIKV RdRp. In order to verify whether the binding of **4w** and NS5 protein is directly related to ZIKV RdRp, we expressed and purified ZIKV RdRp domain, and detected its activity according to the method described in the literature (Qian et al., 2022) (Figure 6A). Through RdRp activity assay results, we found that **4w** inhibited ZIKV RdRp activity with EC_50_ = 11.38 ± 0.51 μM (Figure 6B). Heparin is a protein chelating agent. According to the literature (Sáez-Álvarez et al., 2019), it can significantly inhibited the activity of ZIKV RdRp. Heparin was selected as the positive control drug in this experiment, and the syto9-only group was used as the negative control, the same volume of DMSO as a positive control.

### 2.6 Compound 4w inhibits ZIKV RNA synthesis

**FIGURE 7:**
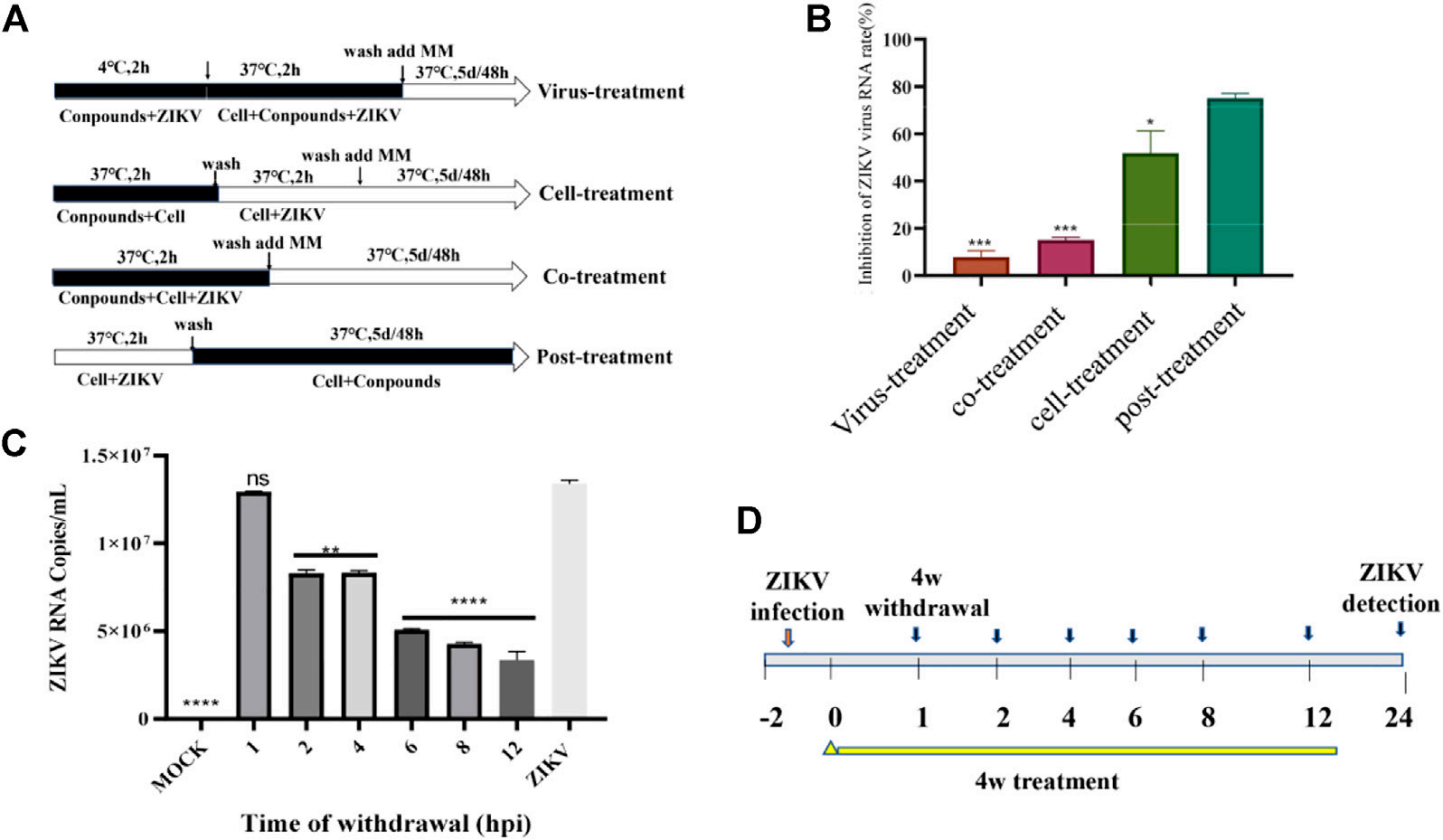
Compound **4w** inhibits the synthesis of ZIKV RNA. **(A)** Flow chart of four different drug treatments; **(B)** Determination of viral load in cell supernatants after four different drug treatments, VS. post-treatment, *p < 0.05; **p < 0.01; ***p < 0.001; ****p < 0.0001; **(C)** experimental procedure of time-sharing withdrawal. **(D)** After drug withdrawal at different time points, the virus load in the cells was measured for 24 h after drug withdrawal.

Through above experiments, we demonstrated that **4w** inhibits ZIKV RdRp activity in a dose-dependent manner. ZIKV RdRp was a important domain in the C-terminal of ZIKV NS5, and plays a crucial role in the replication and synthesis of RNA in the life cycle of ZIKV. In order to further verify that **4w** activity is related to RdRp domain inhibition, we examined the effect of **4w** on the replication cycle of ZIKV using four different drug treatments and time-sharing withdrawal experiments (Figure 7A). Through four different treatments with **4w**, we found that **4w** mainly acts on the post-entry stage of the virus, and it has a significant inhibitory effect on the viral load in cells after virus infection (Figure 7B). In the time of drug withdrawal experiment (Figure 7C), we found that drug withdrawal 4–6 h after dosing could significantly inhibit the release of progeny virus in the supernatant of ZIKV infected cells. From the results of drug treatment, we found that **4w** may mainly act on the ZIKV RNA synthesis stage (Figure 7D), which once again suggesting that **4w** binds into the ZIKV RdRp domain inhibition.

### 2.7 Compound 4w inhibits progeny virus release in different cell lines

**FIGURE 8:**
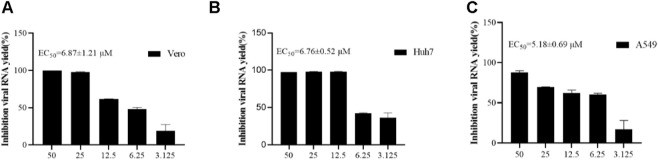
Compound **4w** inhibits virus release of ZIKV progeny in different cell lines. **(A)** Vero cells; **(B)** Huh7 cells; **(C)** A549 cells.

In order to re-verify the inhibitory effect of **4w** on ZIKV at the molecular level, we used ZIKV to infect cell lines of different origins (A549, Vero, Huh7 cells) and then treated with **4w** in a concentration gradient. The change of viral load in the supernatant of cells after treatment was detected by qRT-PCR method. It was found that **4w** could significantly inhibit the replication of viral RNA in different cell lines infected with ZIKV (Figure 8). In Vero, A549 and Huh7 cells, **4w** inhibited the replication of ZIKV RNA with EC_50_ values 6.871 ± 1.21, 5.18 ± 0.69, and 6.76 ± 0.52 μM, respectively. These results indicate that the inhibitory effect of **4w** on ZIKV is not dependent on cell species specificity.

### 2.8 Compound 4w inhibits the expression of ZIKV E and NS5 protein

**FIGURE 9:**
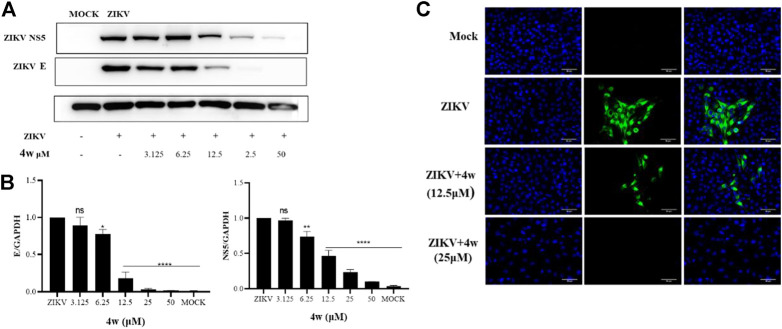
Compound **4w** inhibited the expression of ZIKV E and NS5 protein. **(A)** Western blot detected the inhibitory effect of **4w** on ZIKV E and NS5 protein under the concentration gradient of **4w**; **(B)** Grayscale analysis and statistics of the inhibition of **4w** on ZIKV E and NS5 protein under the concentration gradient of WB detection; Data is the mean (±SD) of three experiments, with DMSO as a positive contro *p < 0.05; **p < 0.01; ***p < 0.001; ****p < 0.0001. **(C)** Detected the inhibitory effect of **4w** on ZIKV E protein by immunofluorescence.

ZIKV E protein is one of the most important structural proteins of ZIKV, which plays an important role in the correct assembly of the virus and evasion of the host’s innate immunity (Jaimipuk et al., 2022). We have verified that **4w** has a significant inhibitory effect on ZIKV-infected cell lines from different sources at the molecular level, but it is unknown whether it has the same antiviral effect at the protein level. Therefore, in order to explore the inhibitory effect of **4w** on ZIKV protein level, we added serially diluted **4w** to ZIKV-infected Vero cells for 48 h, collected the total protein for WB experiments, and then detected the effect of **4w** on ZIKV E and NS5 protein. At the same time, the inhibitory effect of **4w** on ZIKV E protein was verified again by immunofluorescence experiment. Through WB experiment (Figure 9A), we found that **4w** can still well inhibit the expression of ZIKV E and NS5 protein at the viral protein level, and its 12.5 μM can significantly inhibit the expression of ZIKV E and NS5 protein (Figure 9B). Immunofluorescence results showed that **4w** 25 μM completely inhibited the expression of ZIKV E protein (Figure 9C).

## 3 Materials and methods

### 3.1 Chemical general information

Melting points were determined on a WRS-1 digital melting point apparatus and are calibrated. ^1^H NMR and ^13^C NMR spectra were obtained on a Brucker AM 400 MHz spectrometer in the indicated solvents. Chemical shifts are expressed in *δ* units and TMS as internal reference. Mass spectra were taken on an Agilent LC/MSD TOF mass spectrometer. Solvents were reagent quality and, when necessary, were purified and dried by typical methods. Concentration of the reaction solutions involved the use of rotary evaporator (Heidolph) at reduced pressure. TLC was performed on silica gel GF254 for TLC (Shanghai Haohong Biopharmaceutical Technology Co., Ltd., Shanghai, China) and spots were visualized by iodine vapors or by irradiation with UV light (254 nm).

### 3.2 Chemistry synthesis

General procedure A: To a well stirred solution of diethyl ethylmalonate (100 mmol) in anhydrous EtOH (250 ml) was added a solution of KOH (100 mmol) in anhydrous EtOH (250 ml) at room temperature overnight. After removing the solvent, the residue was washed with a small amount of ethyl acetate and suspended in anhydrous CH_3_CN (250 ml), Et_3_N (120 mmol) and MgCl_2_ (120 mmol) were added and the mixtures were stirred at room temperature for 1 h. Then were added the solutions of cyclohexyl acetyl imidazoline, prepared 30 min before by reaction between **1a/b** (100 mmol) and N,N-carbonyldiimidazole (CDI, 50 mmol) in CH_3_CN (200 ml). The reaction mixtures were refluxed for 8–12 h. After the mixture was cooled to rt. Then the CH_3_CN was removed in vacuo and the residues were dissolved in H_2_O and subsequent acidification to pH 6 with 5% aq HCl. The aqueous layer was extracted with EtOAc (3 × 100 ml), and the combined organic layers were washed with saturated NaHCO_3_ (3 × 200 ml) and brine (3 × 200 ml), dried (Na_2_SO_4_), filtered and concentrated to give the crude products **2a** and **2b**, respectively, which were precisely used in the following process without further purification.

General procedure B: Sodium metal (102 mmol) was dissolved in 250 ml of absolute ethanol, and thiourea (102 mmol) and *β*-Ketoesters **2a, 2b** (85 mmol) were added to the clear solution at room temperature. The reaction mixture was refluxed for 8–12 h. After completion of the reaction, the solvent was removed in vacuo and the residues were dissolved in water and were precipitated addition of conc. HCl until pH = 4. The resulting precipitate was filtered off, washed with EtOH, then dried to give **3a, 3b**, which is directly used in the next step without further purification.

General procedure C: To a solution of **5a-m** (40 mmol) in dichloroethane (150 ml) was added Et_3_N (48 mmol) at 0°C, then bromoacetyl bromide (48 mmol) was added dropwise to the well-stirred mixture over a period of 45 min. The reaction solution was extracted with CH_2_Cl_2_ (50 ml × 3), and the combined organic layers were dried with Na_2_SO_4_. The residue was purified by recrystallization (CH_2_Cl_2_/petroleum ether) to give **4a–d**.

General procedure D: Compound **3a/b** (1.2 mmol) were dissolved in DMF (30 ml), and K_2_CO_3_ (1.32 mmol) was added. And then, **6a-m** (1.32 mmol) were added to the reaction solution. The reaction mixture was stirred at room temperature for 6–10 h. After the reaction was completed, the mixture was filtered under reduced pressure and H_2_O (200 ml) was added, then the solution was extracted with EtOAc (50 ml × 3). The combined organic layers were dried with Na_2_SO_4_, filtered, and concentrated. The residue was purified by column chromatography on silica gel using EtOAc/petroleum ether as an eluent to give **4a–z**. Purity of final compounds was verified to be >95% by HPLC analysis.

#### 3.2.1 2-[(4-cyclohexyl-5-ethyl-6-oxo-1,6-dihydropyrimidin-2-yl)thio]-N-(2-methoxyphenyl)acetamide**(4a)**

General procedure D. White solid; yield: 87%; m.p. 187–188°C; ^1^H NMR (400 MHz, DMSO-*d*_*6*_, ppm) δ 0.87–0.91 (m, 1H, cyclohexyl-H), 0.93–0.97 (t, 3H, *J* = 7.4 Hz,−CH_2_CH_3_), 1.17–1.26 (m, 2H, cyclohexyl-H), 1.34–1.42 (m, 2H, cyclohexyl-H), 1.45–1.60 (m, 5H, cyclohexyl-H), 2.33–2.39 (q, 2H, *J* = 7.4 Hz, −CH_2_CH_3_), 2.52–2.60 (m, 1H, cyclohexyl-H), 3.80 (s, 3H, −OCH_3_), 4.06 (s, 2H, S-CH_2_), 6.85–6.90 (m, 1H, Ph-H), 7.00–7.06 (m, 2H, Ph-H), 8.04–8.08 (m, 1H, Ph-H), 9.33 (s, 1H, NH); ESI-MS: m/z [M + H]^+^, 402.1846. C_21_H_27_N_3_O_3_S (401.18).

#### 3.2.2 2-{[4-(cyclohexylmethyl)-5-ethyl-6-oxo-1,6-dihydropyrimidin-2-yl]thio}-N-(2-methoxyphenyl) acetamide **(4b)**

General procedure D. White solid; yield: 85%; m.p. 204–205°C; ^1^H NMR (400 MHz, DMSO-*d*_*6*_,ppm) δ 0.81–1.00 (m, 5H, cyclohexyl-H), 0.95–0.99 (t, 3H, *J* = 6.6 Hz,−CH_2_CH_3_), 1.40–1.50 (m, 5H, cyclohexyl-H), 1.59–1.68 (m, 1H, cyclohexyl-H), 2.29–2.33 (d, 2H, *J* = 6.7 Hz, cyclohexyl-CH_2_), 2.34–2.39 (q, 2H, *J* = 6.4 Hz, −CH_2_CH_3_), 3.78 (s, 3H, −OCH_3_), 4.03 (s, 2H, S-CH_2_), 6.85–6.92 (m, 1H, Ph-H), 6.98–7.06 (m, 2H, Ph-H), 8.03–8.08 (m, 1H, Ph-H), 9.33 (s, 1H, NH); ESI-MS: m/z [M + H]^+^, 416.2002. C_22_H_29_N_3_O_3_S (415.19)

#### 3.2.3 2-[(4-cyclohexyl-5-ethyl-6-oxo-1,6-dihydropyrimidin-2-yl)thio]-N-(3-methoxyphenyl)acetamide **(4c)**

General procedure D. White solid; yield: 88%; m.p. 207–209°C;^1^H NMR (400 MHz, DMSO-*d*_*6*_,ppm) δ 0.86–0.90 (m, 1H, cyclohexyl-H), 0.91–0.95 (t, 3H, *J* = 7.4 Hz, −CH_2_CH_3_), 1.12–1.25 (m, 2H, cyclohexyl-H), 1.30–1.38 (m, 2H, cyclohexyl-H), 1.40–1.60 (m, 5H, cyclohexyl-H), 2.31–2.37 (q, 2H, *J* = 7.5 Hz, −CH_2_CH_3_), 2.51–2.57 (m, 1H, cyclohexyl-H), 3.70 (s, 3H, −OCH_3_), 3.97 (s, 2H, S-CH_2_), 6.58–6.62 (m, 1H, Ph-H), 7.10–7.14 (m, 1H, Ph-H), 7.16–7.20 (m, 1H, Ph-H), 7.31–7.32 (m, 1H, Ph-H), 10.26 (s, 1H, NH), 12.54 (s, 1H, NH); ESI-MS: m/z [M + H]^+^, 402.1846. C_21_H_27_N_3_O_3_S (401.18)

#### 3.2.4 2-{[4-(cyclohexylmethyl)-5-ethyl-6-oxo-1,6-dihydropyrimidin-2-yl]thio}-N-(3-methoxyphenyl) acetamide **(4d)**

General procedure D. White solid; yield: 85%; m.p. 190–193°C; ^1^H NMR (400 MHz, DMSO-*d*_*6*_,ppm) δ 0.74–0.91 (m, 5H, cyclohexyl-H), 0.92–0.96 (t, 3H, *J* = 7.3 Hz, −CH_2_CH_3_), 1.35–1.45 (m, 5H, cyclohexyl-H), 1.57–1.68 (m, 1H, cyclohexyl-H), 2.23–2.27 (d, 2H, *J* = 6.7 Hz,cyclohexyl-CH_2_), 2.29–2.35 (q, 2H, *J* = 7.7 Hz, −CH_2_CH_3_), 3.70 (s, 3H, −OCH_3_), 3.97 (s, 2H, S-CH_2_), 6.60–6.63 (m, 1H, Ph-H), 7.10–7.14 (m, 1H, Ph-H), 7.16–7.22 (m, 1H, Ph-H), 7.31–7.33 (m, 1H, Ph-H), 10.20 (s, 1H, NH), 12.58 (s, 1H, NH); ESI-MS: m/z [M + H]^+^, 416.2002. C_22_H_29_N_3_O_3_S (415.19).

#### 3.2.5 2-[(4-cyclohexyl-5-ethyl-6-oxo-1,6-dihydropyrimidin-2-yl)thio]-N-(4-methoxyphenyl)acetamide **(4e)**

General procedure D. White solid; yield: 86%; m.p. 264–266°C; ^1^H NMR (400 MHz, DMSO-*d*_*6*_,ppm) δ 0.92–0.94 (t, 3H, *J* = 7.1 Hz, -CH_2_CH_3_), 0.95–1.00 (m, 1H, cyclohexyl-H), 1.12–1.27 (m, 2H, cyclohexyl-H), 1.32–1.42 (m, 2H, cyclohexyl-H), 1.43–1.63 (m, 5H, cyclohexyl-H), 2.32–2.38 (q, 2H, *J* = 7.4 Hz, −CH_2_CH_3_), 2.52–2.58 (m, 1H, cyclohexyl-H), 3.70 (s, 3H, −OCH_3_), 3.96 (s, 2H, S-CH_2_), 6.85–6.87 (m, 2H, Ph-H), 7.49–7.51 (m, 2H, Ph-H), 10.10 (s, 1H, NH); ESI-MS: m/z [M + H]^+^, 402.1846. C_21_H_27_N_3_O_3_S (401.18).

#### 3.2.6 2-{[4-(cyclohexylmethyl)-5-ethyl-6-oxo-1,6-dihydropyrimidin-2-yl]thio}-N-(4-methoxyphenyl) acetamide **(4f)**

General procedure D. White solid; yield: 84%; m.p. 212–214°C; ^1^H NMR (400 MHz, DMSO-*d*_*6*_,ppm) δ 0.75–0.90 (m, 2H, cyclohexyl-H), 0.90–0.95 (m, 3H, cyclohexyl-H), 0.94–0.96 (t, 3H, *J* = 7.1 Hz, −CH_2_CH_3_), 1.35–1.52 (m, 5H, cyclohexyl-H), 1.61–1.72 (m, 1H, cyclohexyl-H), 2.27–2.28 (d, 2H, *J* = 7.1 Hz, cyclohexyl-CH_2_), 2.31–2.35 (q, 2H, *J* = 7.4 Hz, −CH_2_CH_3_), 3.70 (s, 3H, −OCH_3_), 3.96 (s, 2H, S-CH_2_), 6.85–6.87 (m, 2H, Ph-H), 7.49–7.51 (m, 2H, Ph-H), 10.06 (s, 1H, NH); ESI-MS: m/z [M + H]^+^, 416.2002. C_22_H_29_N_3_O_3_S(415.19)

#### 3.2.7 2-[(4-cyclohexyl-5-ethyl-6-oxo-1,6-dihydropyrimidin-2-yl)thio]-N-(2-fluorophenyl)acetamide **(4g)**

General procedure D. White solid; yield: 86%; m.p. 206–208°C; ^1^H NMR (400 MHz, DMSO-*d*_*6*_,ppm) δ 0.83–0.90 (m, 1H, cyclohexyl-H), 0.92–0.96 (t, 3H, *J* = 7.4 Hz, −CH_2_CH_3_), 1.13–1.26 (m, 2H, cyclohexyl-H), 1.34–1.42 (m, 2H, cyclohexyl-H), 1.43–1.61 (m, 5H, cyclohexyl-H), 2.32–2.38 (q, 2H, *J* = 7.3 Hz, −CH_2_CH_3_), 2.52–2.58 (m, 1H, cyclohexyl-H), 4.09 (s, 2H, S-CH_2_), 7.06–7.16 (m, 2H, Ph-H), 7.27–7.28 (m, 1H, Ph-H), 8.02–8.08 (m, 1H, Ph-H), 10.06 (s, 1H, NH), 12.54 (s, 1H, NH); ESI-MS: m/z [M + H]^+^, 390.1647. C_20_H_24_FN_3_O_2_S (389.16).

#### 3.2.8 2-{[4-(cyclohexylmethyl)-5-ethyl-6-oxo-1,6-dihydropyrimidin-2-yl]thio}-N-(2-fluorophenyl) acetamide **(4h)**

General procedure D. White solid; yield: 86%; m.p. 180–181°C; ^1^H NMR (400 MHz, DMSO-*d*_*6*_,ppm) δ 0.77–0.93 (m, 5H, cyclohexyl-H), 0.93–0.97 (t, 3H, *J* = 7.3 Hz, −CH_2_CH_3_), 1.39–1.49 (m, 5H, cyclohexyl-H), 1.61–1.70 (m, 1H, cyclohexyl-H), 2.27–2.31 (d, 2H, *J* = 7.3 Hz, cyclohexyl-CH_2_), 2.31–2.36 (q, 2H, *J* = 7.3 Hz, −CH_2_CH_3_), 4.07 (s, 2H, S-CH_2_), 7.08–7.16 (m, 2H, Ph-H), 7.22–7.28 (m, 1H, Ph-H), 7.98–8.03 (m, 1H, Ph-H), 9.98 (s, 1H, NH), 12.58 (s, 1H, NH); ESI-MS: m/z [M + H]^+^, 404.1800. C_21_H_26_FN_3_O_2_S (403.17).

#### 3.2.9 2-[(4-cyclohexyl-5-ethyl-6-oxo-1,6-dihydropyrimidin-2-yl)thio]-N-(3-fluorophenyl)acetamide **(4i)**

General procedure D. White solid; yield: 86%; m.p. 184–187°C; ^1^H NMR (400 MHz, DMSO-*d*_*6*_,ppm) δ 0.78–0.88 (m, 1H, cyclohexyl-H), 0.90–0.94 (t, 3H, *J* = 7.1 Hz, −CH_2_CH_3_), 1.10–1.23 (m, 2H, cyclohexyl-H), 1.30–1.45 (m, 5H, cyclohexyl-H), 1.46–1.58 (m, 2H, cyclohexyl-H), 2.31–2.37 (q, 2H, *J* = 7.1 Hz, −CH_2_CH_3_), 2.50–2.55 (m, 1H, cyclohexyl-H), 3.98 (s, 2H, S-CH_2_), 6.81–6.89 (m, 1H, Ph-H), 7.28–7.34 (m, 2H, Ph-H), 7.56–7.63 (m, 1H, Ph-H), 10.50 (s, 1H, NH), 12.54 (s, 1H, NH); ESI-MS: m/z [M + H]^+^, 390.1645. C_20_H_24_FN_3_O_2_S (389.16).

#### 3.2.10 2-{[4-(cyclohexylmethyl)-5-ethyl-6-oxo-1,6-dihydropyrimidin-2-yl]thio}-N-(3-fluorophenyl) acetamide **(4j)**

General procedure D. White solid; yield: 86%; m.p. 216–218°C; ^1^H NMR (400 MHz, DMSO-*d*_*6*_,ppm) δ 0.73–0.91 (m, 5H, cyclohexyl-H), 0.92–0.96 (t, 3H, *J* = 7.3 Hz,−CH_2_CH_3_), 1.34–1.44 (m, 5H, cyclohexyl-H), 1.55–1.64 (m, 1H, cyclohexyl-H), 2.22–2.26 (d, 2H, *J* = 6.9 Hz, cyclohexyl-CH_2_), 2.28–2.35 (q, 2H, *J* = 7.6 Hz, −CH_2_CH_3_), 3.98 (s, 2H, S-CH_2_), 6.83–6.89 (m, 1H, Ph-H), 7.28–7.36 (m, 2H, Ph-H), 7.58–7.63 (m, 1H, Ph-H), 10.44 (s, 1H, NH), 12.59 (s, 1H, NH); ESI-MS: m/z [M + H]^+^, 404.1802. C_21_H_26_FN_3_O_2_S (403.17).

#### 3.2.11 2-[(4-cyclohexyl-5-ethyl-6-oxo-1,6-dihydropyrimidin-2-yl)thio]-N-(4-fluorophenyl)acetamide **(4k)**

General procedure D. White solid; yield: 85%; m.p. 273–274°C; ^1^H NMR (400 MHz, DMSO-*d*_*6*_,ppm) δ 0.81–0.91 (m, 1H, cyclohexyl-H), 0.91–0.95 (t, 3H, *J* = 7.4 Hz, −CH_2_CH_3_), 1.12–1.23 (m, 2H, cyclohexyl-H), 1.31–1.38 (m, 2H, cyclohexyl-H), 1.41–1.60 (m, 5H, cyclohexyl-H), 2.32–2.37 (q, 2H, *J* = 7.3 Hz, −CH_2_CH_3_), 2.51–2.56 (m, 1H, cyclohexyl-H), 3.97 (s, 2H, S-CH_2_), 7.11–7.15 (m, 2H, Ph-H), 7.61–7.64 (m, 2H, Ph-H), 10.35 (s, 1H, NH), 12.52 (s, 1H, NH); ESI-MS: m/z [M + H]^+^, 390.1646. C_20_H_24_FN_3_O_2_S (389.16).

#### 3.2.12 2-{[4-(cyclohexylmethyl)-5-ethyl-6-oxo-1,6-dihydropyrimidin-2-yl]thio}-N-(4-fluorophenyl) acetamide **(4l)**

General procedure D. White solid; yield: 85%; m.p. 261–262°C; ^1^H NMR (400 MHz, Pyr-*d*_*5*_, ppm) δ 0.87–0.98 (m, 2H, cyclohexyl-H), 1.00–1.10 (m, 3H, cyclohexyl-H), 1.20–1.24 (t, 3H, *J* = 7.3 Hz, −CH_2_CH_3_), 1.47–1.67 (m, 5H, cyclohexyl-H), 1.85–1.96 (m, 1H, cyclohexyl-H), 2.45–2.48 (d, 2H, *J* = 7.3 Hz, cyclohexyl-CH_2_), 2.63–2.68 (q, 2H, *J* = 7.4 Hz, −CH_2_CH_3_), 4.39 (s, 2H, S-CH_2_), 7.14–7.18 (m, 2H, Ph-H), 7.95–7.98 (m, 2H, Ph-H), 11.12 (s, 1H, NH); ESI-MS: m/z [M + H]^+^, 404.1803. C_21_H_26_FN_3_O_2_S (403.17).

#### 3.2.13 2-[(4-cyclohexyl-5-ethyl-6-oxo-1,6-dihydropyrimidin-2-yl)thio]-N-(4-(trifluoromethyl)phenyl) acetamide**(4m)**

General procedure D. White solid; yield: 88%; m.p. 257–259°C; ^1^H NMR (400 MHz, DMSO-*d*_*6*_, ppm) δ 0.72–0.83 (m, 1H, cyclohexyl-H), 0.91–0.94 (t, 3H, *J* = 7.3 Hz,−CH_2_CH_3_), 1.12–1.18 (m, 2H, cyclohexyl-H), 1.29–1.42 (m, 5H, cyclohexyl-H), 1.44–1.55 (m, 2H, cyclohexyl-H), 2.31–2.37 (q, 2H, *J* = 7.2 Hz, −CH_2_CH_3_), 2.51–2.55 (m, 1H, cyclohexyl-H), 4.03 (s, 2H, S-CH_2_), 7.65–7.68 (d, 2H, *J* = 8.3 Hz, Ph-H), 7.81–7.83 (d, 2H, *J* = 8.3 Hz, Ph-H), 10.66 (s, 1H, NH); ESI-MS: m/z [M + H]^+^, 440.1614. C_21_H_24_F_3_N_3_O_2_S (439.15).

#### 3.2.14 2-{[4-(cyclohexylmethyl)-5-ethyl-6-oxo-1,6-dihydropyrimidin-2-yl]thio}-N-[4-(trifluoromethyl) phenyl] acetamide **(4n)**

General procedure D. White solid; yield: 89%; m.p. 206–207°C; ^1^H NMR (400 MHz, DMSO-*d*_*6*_, ppm) δ 0.67–0.88 (m, 5H, cyclohexyl-H), 0.92–0.95 (t, 3H, *J* = 7.1 Hz, −CH_2_CH_3_), 1.27–1.40 (m, 5H, cyclohexyl-H), 1.48–1.60 (m, 1H, cyclohexyl-H), 2.20–2.25 (d, 2H, *J* = 6.5 Hz, cyclohexyl-CH_2_), 2.28–2.34 (q, 2H, *J* = 6.9 Hz, −CH_2_CH_3_), 4.01 (s, 2H, S-CH_2_), 7.65–7.68 (d, 2H, *J* = 8.2 Hz, Ph-H), 7.82–7.84 (d, 2H, *J* = 8.4 Hz, Ph-H), 10.61 (s, 1H, NH), 12.59 (s, 1H, NH); ESI-MS: m/z [M + H]^+^, 454.1767. C_22_H_26_F_3_N_3_O_2_S (453.17).

#### 3.2.15 2-[(4-cyclohexyl-5-ethyl-6-oxo-1,6-dihydropyrimidin-2-yl)thio]-N-[4-(trifluoromethoxy)phenyl] acetamide **(4o)**

General procedure D. White solid; yield: 86%; m.p. 252–254°C; ^1^H NMR (400 MHz, DMSO-*d*_*6*_, ppm) δ 0.70–0.82 (m, 1H, cyclohexyl-H), 0.91–0.95 (t, 3H, *J* = 7.4 Hz, −CH_2_CH_3_), 1.10–1.25 (m, 2H, cyclohexyl-H), 1.30–1.44 (m, 5H, cyclohexyl-H), 1.45–1.55 (m, 2H, cyclohexyl-H), 2.30–2.38 (q, 2H, *J* = 7.2 Hz, -CH_2_CH_3_), 2.50–2.58 (m, 1H, cyclohexyl-H), 3.99 (s, 2H, S-CH_2_), 7.29–7.34 (d, 2H, *J* = 8.8 Hz, Ph-H), 7.71–7.75 (d, 2H, *J* = 8.9 Hz, Ph-H), 10.52 (s, 1H, NH), 12.50 (s, 1H, NH); ESI-MS: m/z [M + H]^+^, 456.1563. C_21_H_24_F_3_N_3_O_3_S (455.15).

#### 3.2.16 2-{[4-(cyclohexylmethyl)-5-ethyl-6-oxo-1,6-dihydropyrimidin-2-yl]thio}-N-[4-(trifluoromethoxy) phenyl] acetamide **(4p)**

General procedure D. White solid; yield: 86%; m.p. 193–195°C; ^1^H NMR (400 MHz, DMSO-*d*_*6*_, ppm) δ 0.70–0.87 (m, 5H, cyclohexyl-H), 0.92–0.96 (t, 3H, *J* = 7.1 Hz, −CH_2_CH_3_), 1.28–1.42 (m, 5H, cyclohexyl-H), 1.51–1.60 (m, 1H, cyclohexyl-H), 2.21–2.27 (d, 2H, *J* = 6.6 Hz, cyclohexyl-CH_2_), 2.28–2.35 (q, 2H, *J* = 7.4 Hz, −CH_2_CH_3_), 3.98 (s, 2H, S-CH_2_), 7.28–7.34 (d, 2H, *J* = 8.4 Hz, Ph-H), 7.70–7.75 (d, 2H, *J* = 8.7 Hz, Ph-H), 10.44 (s, 1H, NH), 12.58 (s, 1H, NH); ESI-MS: m/z [M + H]^+^, 470.1720. C_22_H_26_F_3_N_3_O_3_S (469.16)

#### 3.2.17 2-[(4-cyclohexyl-5-ethyl-6-oxo-1,6-dihydropyrimidin-2-yl)thio]-N-[3-(trifluoromethoxy)phenyl] acetamide **(4q)**

General procedure D. White solid; yield: 85%; m.p. 219–221°C; ^1^H NMR (400 MHz, DMSO-*d*_*6*_, ppm) δ 0.70–0.82 (m, 1H, cyclohexyl-H), 0.90–0.94 (t, 3H, *J* = 7.3 Hz, −CH_2_CH_3_), 1.07–1.21 (m, 2H, cyclohexyl-H), 1.28–1.42 (m, 5H, cyclohexyl-H), 1.43–1.55 (m, 2H, cyclohexyl-H), 2.31–2.36 (q, 2H, *J* = 7.3 Hz, −CH_2_CH_3_), 2.51–2.55 (m, 1H, cyclohexyl-H), 3.99 (s, 2H, S-CH_2_), 6.98–7.03 (m, 1H, Ph-H), 7.39–7.45 (m, 1H, Ph-H), 7.50–7.54 (m, 1H, Ph-H), 7.78–7.79 (m, 1H, Ph-H), 10.60 (s, 1H, NH), 12.56 (s, 1H, NH); ESI-MS: m/z [M + H]^+^, 456.1563. C_21_H_24_F_3_N_3_O_3_S (455.15).

#### 3.2.18 2-{[4-(cyclohexylmethyl)-5-ethyl-6-oxo-1,6-dihydropyrimidin-2-yl]thio}-N-[3-(trifluoromethoxy) phenyl] acetamide **(4r)**

General procedure D. White solid; yield: 86%; m.p. 180–182°C; ^1^H NMR (400 MHz, DMSO-*d*_*6*_, ppm) δ 0.70–0.88 (m, 5H, cyclohexyl-H), 0.92–0.96 (t, 3H, *J* = 7.0 Hz, −CH_2_CH_3_), 1.28–1.41 (m, 5H, cyclohexyl-H), 1.52–1.63 (m, 1H, cyclohexyl-H), 2.21–2.27 (d, 2H, *J* = 6.5 Hz, cyclohexyl-CH_2_), 2.27–2.34 (q, 2H, *J* = 7.3 Hz, −CH_2_CH_3_), 3.99 (s, 2H, S-CH_2_), 6.99–7.04 (m, 1H, Ph-H), 7.39–7.45 (m, 1H, Ph-H), 7.49–7.54 (m, 1H, Ph-H), 7.79–7.80 (m, 1H, Ph-H), 10.54 (s, 1H, NH), 12.60 (s, 1H, NH); ESI-MS: m/z [M + H]^+^, 470.1720. C_22_H_26_F_3_N_3_O_3_S (469.16).

#### 3.2.19 2-[(4-cyclohexyl-5-ethyl-6-oxo-1,6-dihydropyrimidin-2-yl)thio]-N-(2,4-difluorophenyl)acetamide **(4s)**

General procedure D. White solid; yield: 84%; m.p. 227–229°C; ^1^H NMR (400 MHz, DMSO-*d*_*6*_, ppm) δ 0.82–0.91 (m, 1H, cyclohexyl-H), 0.92–0.95 (t, 3H, *J* = 7.1 Hz, −CH_2_CH_3_), 1.15–1.29 (m, 2H, cyclohexyl-H), 1.36–1.43 (m, 2H, cyclohexyl-H), 1.45–1.60 (m, 5H, cyclohexyl-H), 2.31–2.39 (q, 2H, *J* = 7.6 Hz, −CH_2_CH_3_), 2.53–2.61 (m, 1H, cyclohexyl-H), 4.08 (s, 2H, S-CH_2_), 7.01–7.09 (m, 1H, Ph-H), 7.28–7.38 (m, 1H, Ph-H), 7.96–8.04 (m, 1H, Ph-H), 10.10 (s, 1H, NH), 12.57 (s, 1H, NH); ESI-MS: m/z [M + H]^+^, 408.1552. C_20_H_23_F_2_N_3_O_2_S (407.15).

#### 3.2.20 2-{[4-(cyclohexylmethyl)-5-ethyl-6-oxo-1,6-dihydropyrimidin-2-yl]thio}-N-(2,4-difluorophenyl) acetamide **(4t)**

General procedure D. White solid; yield: 85%; m.p. 192–194°C; ^1^H NMR (400 MHz, DMSO-*d*_*6*_,ppm) δ 0.78–0.89 (m, 2H, cyclohexyl-H), 0.90–1.02 (m, 3H, cyclohexyl-H), 0.93–0.97 (t, 3H, *J* = 7.0 Hz, −CH_2_CH_3_), 1.41–1.52 (m, 5H, cyclohexyl-H), 1.60–1.70 (m, 1H, cyclohexyl-H), 2.27–2.30 (d, 2H, *J* = 6.9 Hz, cyclohexyl-CH_2_), 2.31–2.36 (q, 2H, *J* = 7.3 Hz, −CH_2_CH_3_), 4.06 (s, 2H, S-CH_2_), 7.01–7.09 (m, 1H, Ph-H), 7.28–7.37 (m, 1H, Ph-H), 7.91–8.00 (m, 1H, Ph-H), 10.01 (s, 1H, NH), 12.57 (s, 1H, NH); ESI-MS: m/z [M + H]^+^, 422.1703. C_21_H_25_F_2_N_3_O_2_S (421.16).

#### 3.2.21 2-[(4-cyclohexyl-5-ethyl-6-oxo-1,6-dihydropyrimidin-2-yl)thio]-N-(2,5-difluorophenyl)acetamide **(4u)**

General procedure D. White solid; yield: 83%; m.p. 190–192°C; ^1^H NMR (400 MHz, DMSO-*d*_*6*_, ppm) δ 0.85–0.91 (m, 1H, cyclohexyl-H), 0.92–0.96 (t, 3H, *J* = 7.4 Hz, −CH_2_CH_3_), 1.16–1.28 (m, 2H, cyclohexyl-H), 1.36–1.43 (m, 2H, cyclohexyl-H), 1.46–1.61 (m, 5H, cyclohexyl-H), 2.32–2.39 (q, 2H, *J* = 7.3 Hz, −CH_2_CH_3_), 2.52–2.61 (m, 1H, cyclohexyl-H), 4.08 (s, 2H, S-CH_2_), 7.02–7.08 (m, 1H, Ph-H), 7.29–7.36 (m, 1H, Ph-H), 7.96–8.03 (m, 1H, Ph-H), 10.10 (s, 1H, NH), 12.51 (s, 1H, NH); ESI-MS: m/z [M + H]^+^, 408.1552. C_20_H_23_F_2_N_3_O_2_S (407.15).

#### 3.2.22 2-{[4-(cyclohexylmethyl)-5-ethyl-6-oxo-1,6-dihydropyrimidin-2-yl]thio}-N-(2,5-difluorophenyl) acetamide **(4v)**

General procedure D. White solid; yield:82%; m.p. 186–188°C; ^1^H NMR (400 MHz, DMSO-*d*_*6*_, ppm) δ 0.79–1.00 (m, 5H, cyclohexyl-H), 0.93–0.97 (t, 3H, *J* = 7.3 Hz, −CH_2_CH_3_), 1.40–1.51 (m, 5H, cyclohexyl-H), 1.60–1.71 (m, 1H, cyclohexyl-H), 2.26–2.30 (d, 2H, *J* = 6.8 Hz, cyclohexyl-CH_2_), 2.31–2.36 (q, 2H, *J* = 7.3 Hz, −CH_2_CH_3_), 4.05 (s, 2H, S-CH_2_), 7.03–7.08 (m, 1H, Ph-H), 7.31–7.36 (m, 1H, Ph-H), 7.91–7.99 (m, 1H, Ph-H), 10.01 (s, 1H, NH), 12.59 (s, 1H, NH); ESI-MS: m/z [M + H]^+^, 422.1708. C_21_H_25_F_2_N_3_O_2_S (421.16).

#### 3.2.23 2-{[4-cyclohexyl-5-ethyl-6-oxo-1,6-dihydropyrimidin-2-yl]thio}-N-(3,5-difluorophenyl)acetamide **(4w)**

General procedure D. White solid; yield:86%; m.p. 238–240°C; ^1^H NMR (400 MHz, Pyr-*d*_*5*_, ppm) δ 1.00–1.10 (m, 1H, cyclohexyl-H), 1.15–1.19 (t, 3H, *J* = 7.4 Hz, −CH_2_CH_3_), 1.22–1.34 (m, 2H, cyclohexyl-H), 1.54–1.68 (m, 5H, cyclohexyl-H), 1.75–1.87 (m, 2H, cyclohexyl-H), 2.64–2.69 (q, 2H, *J* = 7.4 Hz, −CH_2_CH_3_), 2.70–2.78 (m, 1H, cyclohexyl-H), 4.38 (s, 2H, S-CH_2_), 6.79–6.86 (m, 1H, Ph-H), 7.68–7.75 (m, 2H, Ph-H), 11.64 (s, 1H, NH); ESI-MS: m/z [M + H]^+^, 408.1549. C_20_H_23_F_2_N_3_O_2_S (407.15).

#### 3.2.24 2-{[4-(cyclohexylmethyl)-5-ethyl-6-oxo-1,6-dihydropyrimidin-2-yl]thio}-N-(3,5-difluorophenyl) acetamide **(4x)**

General procedure D. White solid; yield:86%; m.p. 214–216°C; ^1^H NMR (400 MHz, DMSO-*d*_*6*_, ppm) δ 0.71–0.89 (m, 5H, cyclohexyl-H), 0.92–0.96 (t, 3H, *J* = 7.3 Hz, −CH_2_CH_3_), 1.34–1.45 (m, 5H, cyclohexyl-H), 1.51–1.60 (m, 1H, cyclohexyl-H), 2.21–2.26 (d, 2H, *J* = 7.2 Hz, cyclohexyl-CH_2_), 2.28–2.34 (q, 2H, *J* = 7.5 Hz, −CH_2_CH_3_), 3.98 (s, 2H, S-CH_2_), 6.87–6.93 (m, 1H, Ph-H), 7.31–7.34 (m, 2H, Ph-H), 10.62 (s, 1H, NH); ESI-MS: m/z [M + H]^+^, 422.1708. C_21_H_25_F_2_N_3_O_2_S (421.16).

#### 3.2.25 2-[(4-cyclohexyl-5-ethyl-6-oxo-1,6-dihydropyrimidin-2-yl)thio]-N-(5-fluoro-2-methoxyphenyl) acetamide **(4y)**

General procedure D. White solid; yield:87%; m.p. 166–167°C; ^1^H NMR (400 MHz, DMSO-*d*_*6*_, ppm) δ 0.79–0.89 (m, 1H, cyclohexyl-H), 0.92–0.95 (t, 3H, *J* = 7.3 Hz, −CH_2_CH_3_), 1.13–1.25 (m, 2H, cyclohexyl-H), 1.32–1.40 (m, 2H, cyclohexyl-H), 1.42–1.55 (m, 5H, cyclohexyl-H), 2.32–2.38 (q, 2H, *J* = 7.6 Hz, −CH_2_CH_3_), 2.52–2.58 (m, 1H, cyclohexyl-H), 4.08 (s, 2H, S-CH_2_), 6.82–6.87 (m, 1H, Ph-H), 6.99–7.04 (m, 1H, Ph-H), 8.00–8.04 (m, 1H, Ph-H), 9.55 (s, 1H, NH), 12.57 (s, 1H, NH); ESI-MS: m/z [M + H]^+^, 420.1752. C_21_H_26_FN_3_O_3_S (419.17).

#### 3.2.26 2-{[4-(cyclohexylmethyl)-5-ethyl-6-oxo-1,6-dihydropyrimidin-2-yl]thio}-N-(5-fluoro-2-methoxy phenyl)acetamide **(4z)**

General procedure D. White solid; yield:88%; m.p. 182–183°C; ^1^H NMR (400 MHz, DMSO-*d*_*6*_,ppm) δ 0.77–0.93 (m, 5H, cyclohexyl-H), 0.95–0.99 (t, 3H, *J* = 7.3 Hz, −CH_2_CH_3_), 1.38–1.49 (m, 5H, cyclohexyl-H), 1.56–1.66 (m, 1H, cyclohexyl-H), 2.28–2.31 (d, 2H, *J* = 6.7 Hz, cyclohexyl-CH_2_), 2.32–2.38 (q, 2H, *J* = 7.3 Hz, −CH_2_CH_3_), 3.80 (s, 3H, −OCH_3_), 4.06 (s, 2H, S-CH_2_), 6.83–6.90 (m, 1H, Ph-H), 7.00–7.06 (m, 1H, Ph-H), 7.99–8.04 (m, 1H, Ph-H), 9.51 (s, 1H, NH), 12.63 (s, 1H, NH); ESI-MS: m/z [M + H]^+^, 434.1908. C_22_H_28_FN_3_O_3_S (433.18).

## 4 Biology assay

### 4.1 Biological experimental materials

African green monkey kidney (Vero) cells, human hepatoma (Huh-7) cells and human alveolar basal epithelial (A549) cells were cultured in Dulbecco’s modified Eagle’s medium (DMEM; Thermo Fisher, United States) supplemented with 10% fetal bovine serum (FBS; Thermo Fisher, United States) at 37°C in 5% CO_2_ incubator. *Aedes albopictus* (C6/36) cells was used to amplify ZIKV and cells were cultured in Roswell Park Memorial Institute medium (RPMI1640; Thermo Fisher, United States) contain 10% FBS. The ZIKV SZ-WIV01 strain (GenBank: KU963796) was kindly donated by Prof. Bo Zhang (Wuhan Institute of Virology, Chinese Academy of Sciences, Wuhan, China).

ZIKV NS5 RdRp Plasmid was a kindly gift from Prof. Shi Yi (Institute of Microbiology, Chinese Academy of Sciences, Beijing, China). Electro-competent *Escherichia coli* BL21 (DE3)-April cells were obtained from Tsingke Biotechnology. The following reagents were purchased from Sigma: NaCl, MgCl_2_, ZnCl_2_, glycerol, imidazole, isopropyl-β-D-1-thiogalactopyranoside (IPTG), 1,4-dithiothreitol (DTT), LB medium (powder), ampicillin (used at 100 μg/ml) and MnCl_2_, ammonium acetate, Tris base. SYTOTM nine green fuorescent dye was obtained from Thermo Fisherand His Pur TM Ni-NTA resin was purchased from GE. Tween 20, heparin, EDTA and oligonucleotides were also purchased from Sigma. ssRNA polyuridylic acid (poly-U) was obtained from Shanghai Yuanye Bio-Technology, ATP were purchased from Armresco. EDTA and oligonucleotides were also purchased from Sigma-Aldrich.

### 4.2 Plaque assay

Vero cells were inoculated in a 12-well plate at 3 × 10^5^ cells/ml and cultured overnight, the medium was discarded and cells were washed with PBS, then the ZIKV (MOI = 0.5) was added to infect at 37°C for 2 h. The cells were washed with PBS, and then a mixture of DMEM supplemented with 4% FBS and 2% low-melting agarose (Amresco, United States) was overlaid on the cells. Next, the cells were fixed with 4% paraformaldehyde for 15 min at 5d post-infection (hpi), washed twice with PBS and stained with 0.8% crystal violet for 10 min. Finally, an enzyme-linked fluorescence spot spectrometer (CTL, United States) was used for image acquisition. The compounds exhibiting more than 50% plaque inhibition at 50 μmol L^−1^ concentration were considered to have anti-ZIKV activity and will be submitted for further anti-ZIKV testing.

### 4.3 Cytotoxicity assay

MTT assay was used to detect the toxicity of the compound. Cells were seeded in 96-well plates and cultured overnight. Compounds with different concentrations were added and incubated for 96 h. Then, 20 μl of 5 mg/ml MTT (Sigma-Aldrich, United States) solution was added to each well and kept at 37°C for 4 h. Subsequently, 100 μl of 12% SDS-50% DMF (Sigma-Aldrich, United States) solution was added and incubated overnight at 37°C. After the crystalline formamidine was completely dissolved, the optical density (OD) value was measured using a microplate reader (BioTek, United States) with a wavelength of 570 nm and a reference wavelength of 630 nm.

### 4.4 Cell thermal shift assay

Vero cells were inoculated in a 10 cm cell culture dish and cultured overnight, and after infection with ZIKV (MOI ≈2), maintenance medium was added to continue culturing for 3 days. Detergent-free protein lysis buffer (25 mM HEPES, 20 mM MgCl_2_, 2 mM DTT) was added, and cells were lysed by sonication. The cell supernatant was collected by centrifugation at 20,000 g for 20 min at 4°C, and the supernatant was divided into two parts, one was added with 50 μM **4w**, and the other was added with the same volume of DMSO as a control. After mixing, the reaction was carried out at room temperature for 60 min. Divide the reacted samples into nine equal aliquots into PCR tubes, 50 μl per tube. The protein was heated in a temperature gradient for 3 min on the PCR machine, and immediately cooled on ice for 3 min. Then centrifuged at 20,000 × g at 4°C for 20 min. The supernatants were collected to prepare the samples, and the binding of **4w** and ZIKV NS5 protein was analyzed by Western blotting (Chen et al., 2020; Chen et al., 2021).

### 4.5 Four different methods of drug treatment

As mentioned above (Duan et al., 2019; Zou et al., 2020), according to the different life cycles of the ZIKV, four different drug treatment methods are set: virus treatment, co-treatment, pre-treatment, post-treatment. For virus treatment, the ZIKV and **4w** were pre-treated in a 4°C refrigerator for 1 h, then the treated virus was added to the cells for 2 h of infection, and the virus was discarded, washed twice with PBS, and added to maintenance medium (containing 2% FBS DMEM high sugar medium), continue to culture at 37°C, 5% CO_2_ incubator for 3 days, collect the cell supernatant for RT-PCR and plaque experiments. For co-treatment: add **4w** and ZIKV to the cells at the same time, incubate at 37°C, 5% CO_2_ for 2 h, then remove the virus and compounds, add maintenance medium for 3 days. Pre-treatment: Compound **4w** and cells were treated at 4°C for 1 h, then **4w** was removed, ZIKV was added for 2 h, remove virus and the maintenance medium was added to continue culturing for 3 days. Post-treatment: The virus was added to the cells to infect for 2 h, remove the virus and **4w** was added to continue culturing for 3 days.

### 4.6 Time of drug withdrawal

The virus was added to the cells and adsorbed for 2 h at 4°C, then the unabsorbed virus was washed away with pre-cooled PBS, and 50 μM **4w** was added at the same time. Withdraw the drug at 1, 2, 4, 6, 8, and 12 h after dosing. The cell supernatant was collected for viral load detection after the maintenance medium continued to culture for 24 h.

### 4.7 *In vitro* ZIKV RdRp activity assay

The ZIKV RdRp was expressed and purified, and the protein expression and purification method was as described before (Xu et al., 2017; Sáez-Álvarez et al., 2019). **4w** was diluted with protein dialysis buffer to 1,500 nM and stored for later use. *In vitro* RdRp polymerase activity assay was described previously (Qian et al., 2022), 1,500 ng of purified RdRp protein, gradient diluted **4w** and positive control drug 5 μM, 2 mg/ml Poly-U, 20mM ATP, 1M Tris-HCl, 100 mg/ml BSA, 100mM MnCl_2_ was added to a 96-well PCR plate. After mixing, the reaction was performed at 30°C in the dark for 60 min. The reaction was terminated with 25 mM EDTA, and then 10 μM Syto9 was added to react at room temperature for 5 min. RdRp protein activity was detected on a real-time fluorescence quantitative PCR instrument.

### 4.8 Quantitative real-time polymerase chain reaction

Cells were infected with ZIKV SZ-WIV01 and then treated with **4w**. The viral load in the supernatant was detected by Quantitative real-time polymerase chain reaction (qRT-PCR) at in different time periods after infection. Then the RNA from culture supernatant was extracted using the *EasyPure* ^®^ Viral DNA/RNA Kit (TransGen Biotech, China) following the manufacturer’s instructions. Intracellular RNA extraction was according to trizol method. A one-step qRT-PCR kit RNA-direct™ Realtime PCR Master Mix (TOYOBO, Japan) and TaqMan probe were used to quantify the viral RNA produced. The primers (NS5 ZIKV 1086F: 5′-CCG​CTG​CCC​AAC​ACA​AG-3′ and NS5 ZIKV 1086R: 3′-TACAGACGTTTTCTT GCAATCAC C-5′) and probe (5′-FAM-AGCCTACCTTGACAAGCAGTCAGACACTCAA-TAMRA -3′) were used for amplification of the ZIKV NS5 region. A standard curve of the serial dilutions was used to quantify the viral RNA yield.

### 4.9 Western blot analysis

Vero cells were infected with ZIKV (MOI = 1) at 37°C for 2 h. Next, the different amounts of **4w** were added to continue culturing for 48 h to extract the total protein in the cells. The total protein was separated in sodium dodecyl sulfate polyacrylamide gel electrophoresis (SDS-PAGE). Subsequently, the protein was transferred to a polyvinylidene fluoride (PVDF) membrane and incubated with ZIKV E (1:2,000) and NS5 antibody (1:2,000) overnight at 4°C (Sino Biological, China) then add the corresponding secondary antibody and incubate at room temperature for 2 h. A specific signal was presented with a chemiluminescent substrate. The antibody signal was recorded and quantified using the Tanon-5200 Multi Imaging System.

### 4.10 Immunofluorescence assay

Vero cells were seeded at 2 × 10^5^ cells/ml in a 24-well plate with cell slides, and 500 μl/well was cultured overnight in a 37°C, 5% CO_2_ incubator. ZIKV (MOI = 1) was added to infect at 37°C for 2 h, and then discarded the virus. The cells were washed twice with PBS and then added to 500 μl of **4w** diluted in concentration gradients. At the same time, virus-only as a positive control and a medium-only as negative control were set. After culturing at 37°C for 72 h, the cell culture supernatant was discarded, and 4% paraformaldehyde was added to fix the cells for 15 min at room temperature. Cells were washed twice with PBS, PBS containing 0.1% TritonX-100 was added to permeabilize cells at 4°C for 5 min, then the cells were washed with PBS for 5 min, and 5% BSA was added to block for 30 min at room temperature. The cells were stained overnight at 4°C using ZIKV E antibody (Sino Biological, China). Then, the Alexa Fluor®CY5 was added for 1 h in the dark. DAPI staining was used to delineate the nucleus of cells. Microscopic examinations were performed on a Leica DMI4000B Microsystem (Wetzlar, Germany), and an image was recorded using the system.

### 4.11 Statistical analysis

The 50% effective concentration (EC_50_) and 50% cytotoxic concentration (CC_50_) of the compound were calculated according to the Reed and Muench method, inhibition rate of ZIKV RNA yield- [(1-drug treatment/positive control) × 100]. The data and graphs were processed using GraphPad Prism8 software, expressed as mean ± standard deviation (Mean ± SD), comparison between groups using ANOVA, *p* < 0.05 was considered statistically significant.

### 4.12 Molecular docking

Molecular docking method was used to detect the interaction between 4w and ZIKV RdRp protein. Download the ZIKV NS5 RdRp protein from the PDB database (PDB ID: 5U0C) (Zhao et al., 2017), and save the protein structure file in pdb format for later use. The **4w** structure was drawn with ChemDraw, converted to 3D format with Chem3D, and saved as mol2 format for future use. Autodock vina1.2.0 was used for molecular docking of compound and protein. The protein and ligand were prepared using the utilities implemented by AutoDockTools1.5.4. The protein was added to polar hydrogen atoms. Gasteiger charges were assigned to both protein and ligand. The region of interest used by Autodock Vina was defined by considering the Zinc ion as the center of a grid box of 10 Å in the *x*, *y*, and *z* directions. The exhaustiveness parameter was set to 10 and the Energy_range to 4, whereas for all other parameters, Autodock Vina defaults were used. Only top-score binding pose was used in subsequent analyses. Subsequent visualization was performed with Pymol.

## 5 Conclusion

The ZIKV RdRp domain was in the C-terminal of ZIKV NS5, which has become an attractive target in anti-ZIKV virus research in recent years due to its conserved sequence and no human homologue. The development of antiviral drugs targeting ZIKV RdRp provides a new idea for the development of effective anti-ZIKV drugs. In this study, 26 acetylarylamine-*S*-DABOs derivatives were designed, synthesized and evaluated against ZIKV replication and infection. It is discovered that eight compounds exhibit potent anti-ZIKV activity. Preliminary SAR analysis showed that the anti-ZIKV activity of these compounds was related to the type, and substitution position of substituent R on *C*-2 terminal benzene ring. Moreover, the length (n) of the connecting carbon chain between the *C*-6 site of the pyrimidine ring and cyclohexyl also affects the activity of the compounds, and there may be a synergistic relationship between n and substituent R. Furthermore, compound **4w** with the best anti-ZIKV activity was subjected to further analysis to detect the antiviral effects and elucidate the target and the mode of action. The results discovered that **4w** targeting ZIKV RdRp, and verified its potent anti-ZIKV activity at the molecular and protein levels. In the process of protein target verification, the accuracy of using ZIKV RdRp as the main drug target was confirmed from many aspects. In order to verify that this antiviral effect has nothing to do with the specificity of cell species, we used cell lines from different sources such as A549, Huh7, Vero to verify its antiviral activity and cytotoxicity, and finally proved that **4w** is a new and has a good antiviral effect compound targeting the ZIKV NS5 RdRp. Although it has not been studied whether it has the same antiviral effect *in vivo*, this study also confirmed that **4w** is a valuable lead for anti-ZIKV drug discovery targeting ZIKV RdRp. On the basis of the current promising structural core element of the active acetylarylamine-*S*-DABOs derivatives, we will further optimize their structures through rational drug design to discover highly potent ZIKV inhibitors.

## Data Availability

The datasets presented in this study can be found in online repositories. The names of the repository/repositories and accession number(s) can be found in the article/Supplementary Material.
