# Supplementary material for: Identification of 6ω-cyclohexyl-2-(phenylamino carbonylmethylthio)pyrimidin-4(3H)-ones targeting the ZIKV NS5 RNA dependent RNA polymerase
Source: Front Chem. 2022 Oct 12;10:1010547. doi: 10.3389/fchem.2022.1010547 (PMC9605737; doi:10.3389/fchem.2022.1010547)
Supplement: Supplementary file 1 [file DataSheet1.PDF]

Supplementary Material

Supplementary Data

^{1}H NMR spectra

4a

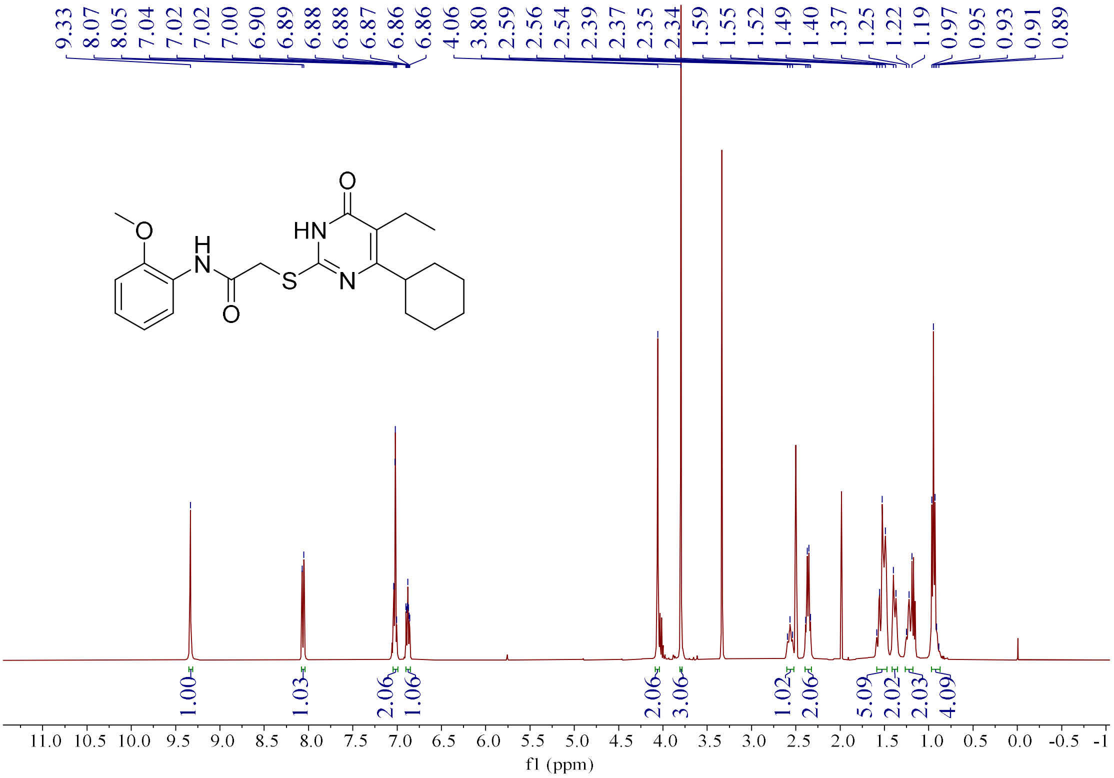

4b

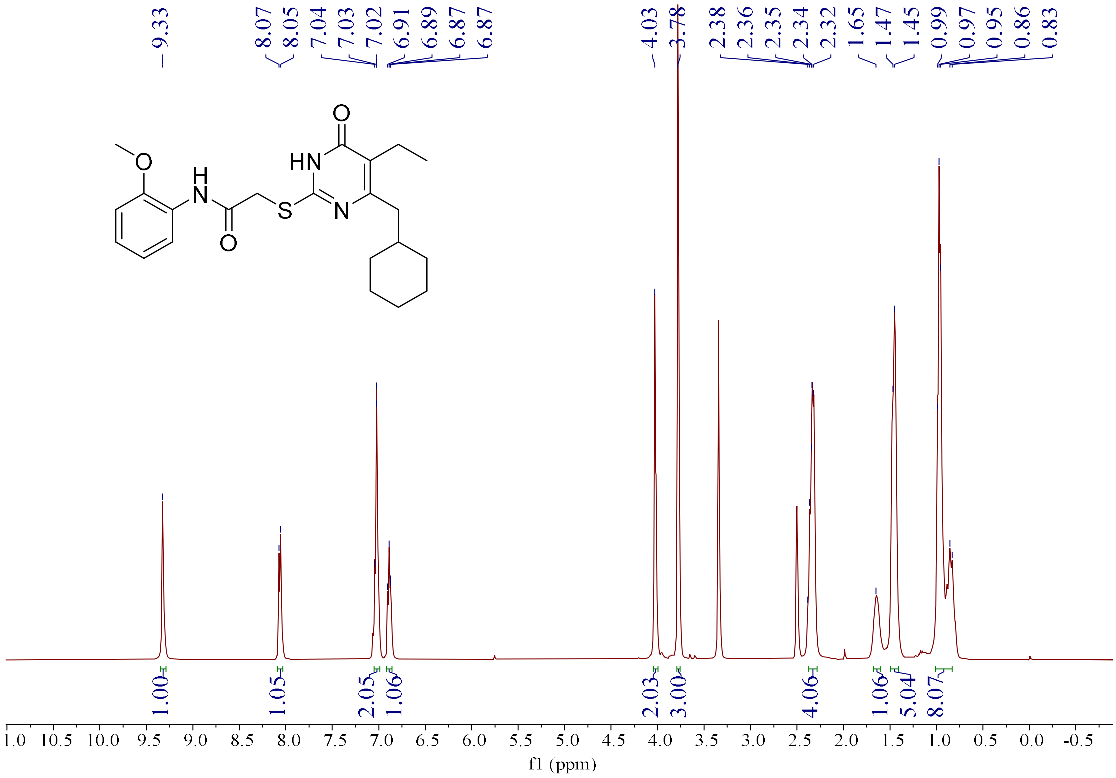

9.33
8.07 8.05 7.04 7.03 7.02 6.91 6.89 6.87 6.87
4.03 3.78
2.38 2.36 2.35 2.34 2.32 1.65 1.47 1.45 0.99 0.97 0.95 0.86 0.83
1.00 1.05 2.05 1.06 2.03 3.00 4.06 1.06 5.04 8.07
f1 (ppm)

4c

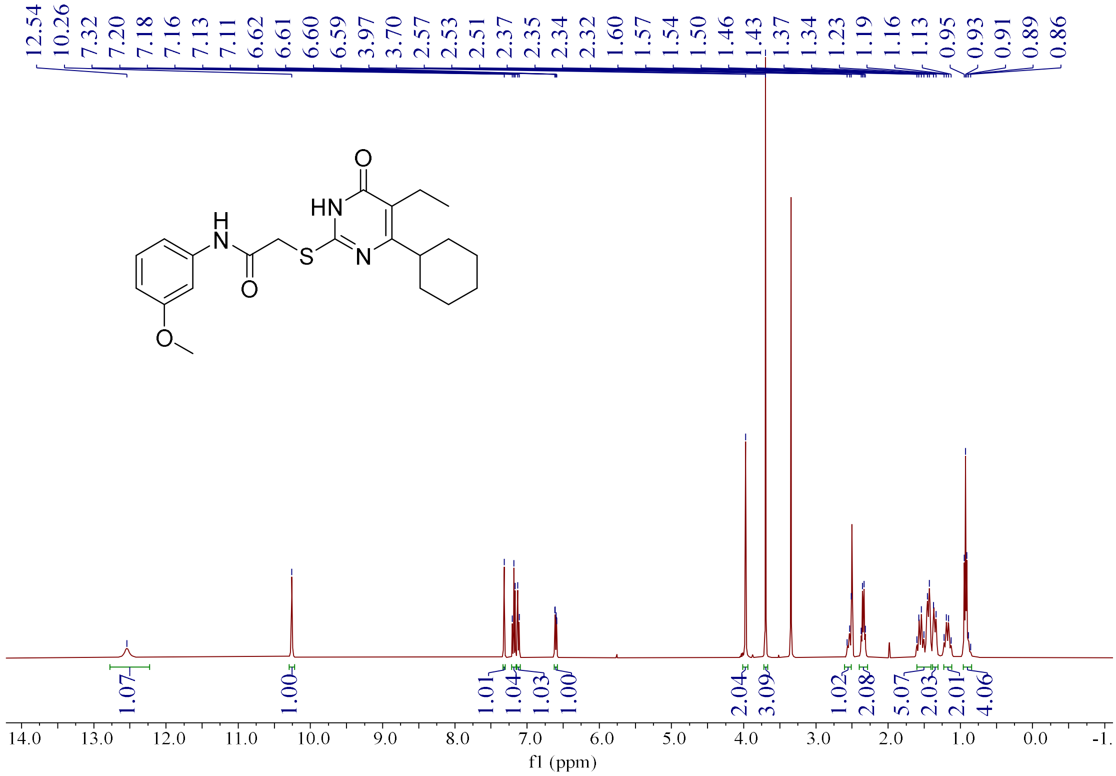

12.54 10.26 7.32 7.20 7.18 7.16 7.13 7.11 6.62 6.61 6.60 6.59 3.97 3.70 2.57 2.53 2.51 2.37 2.35 2.34 2.32 1.60 1.57 1.54 1.50 1.46 1.43 1.37 1.34 1.23 1.19 1.16 1.13 0.95 0.93 0.91 0.89 0.86
1.07 1.00 1.01 1.04 1.03 1.00 2.04 3.09 1.02 2.08 5.07 2.03 2.01 4.06
f1 (ppm)

4d

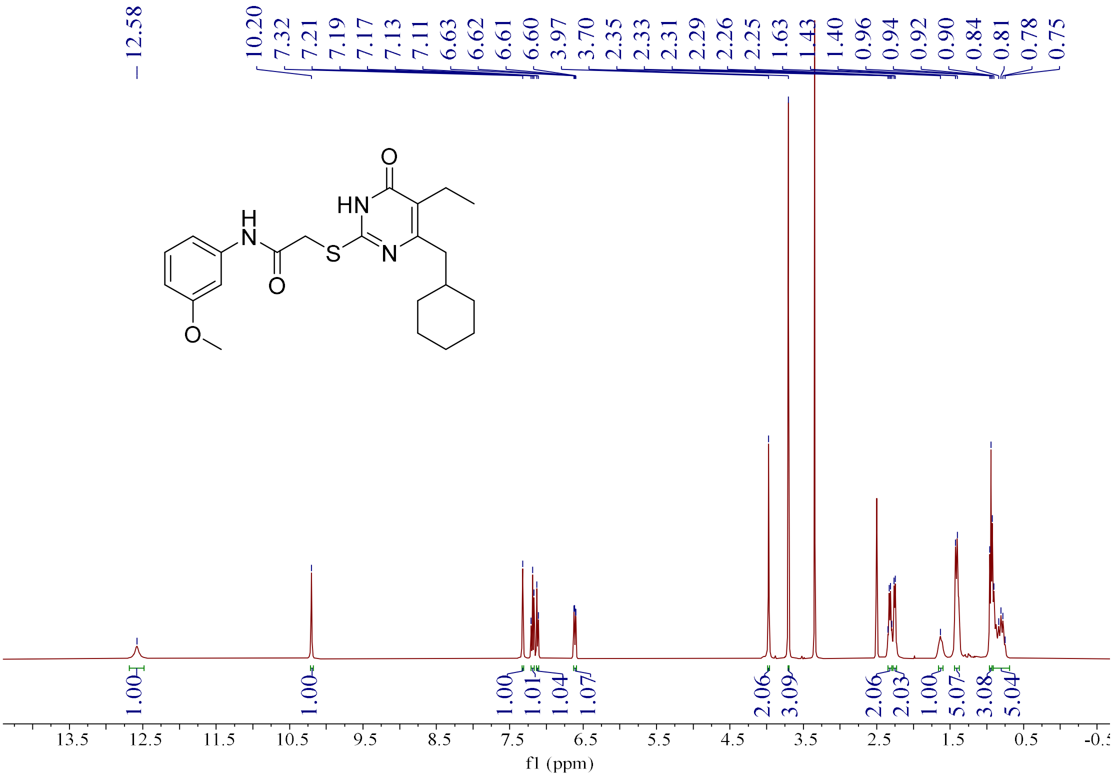
12.58
10.20 7.32 7.21 7.19 7.17 7.13 7.11 6.63 6.62 6.61 6.60 3.97 3.70 2.35 2.33 2.31 2.29 2.26 2.25 1.63 1.43 1.40 0.96 0.94 0.92 0.90 0.84 0.81 0.78 0.75
HN
H
N
O
S
N
O
1.00 1.00 1.00 1.01 1.04 1.07 2.06 3.09 2.06 2.03 1.00 5.07 3.08 5.04
13.5 12.5 11.5 10.5 9.5 8.5 7.5 6.5 5.5 4.5 3.5 2.5 1.5 0.5
f1 (ppm)

4e

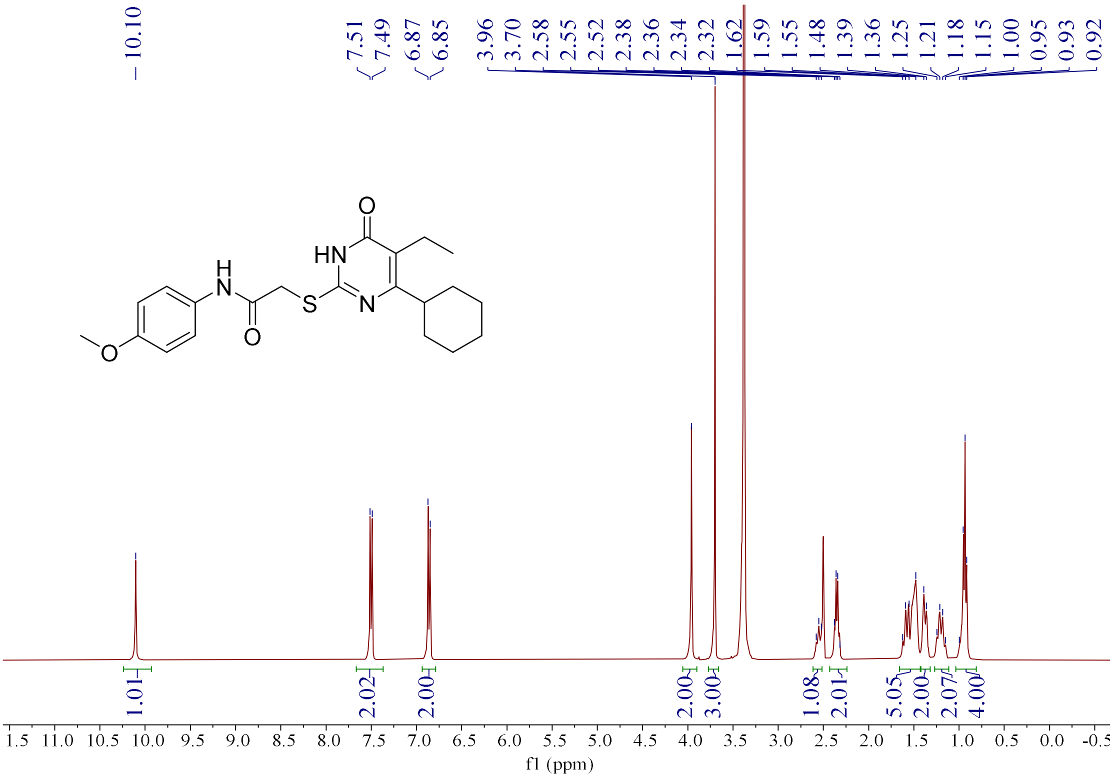
10.10
7.51 7.49 6.87 6.85
3.96 3.70 2.58 2.55 2.52 2.38 2.36 2.34 2.32 1.62 1.59 1.55 1.48 1.39 1.36 1.25 1.21 1.18 1.15 1.00 0.95 0.93 0.92
HN
H
N
O
S
N
O
1.01 2.02 2.00 2.00 3.00 1.08 2.01 5.05 2.00 2.07 4.00
11.0 10.5 10.0 9.5 9.0 8.5 8.0 7.5 7.0 6.5 6.0 5.5 5.0 4.5 4.0 3.5 3.0 2.5 2.0 1.5 1.0 0.5 0.0
f1 (ppm)

4f

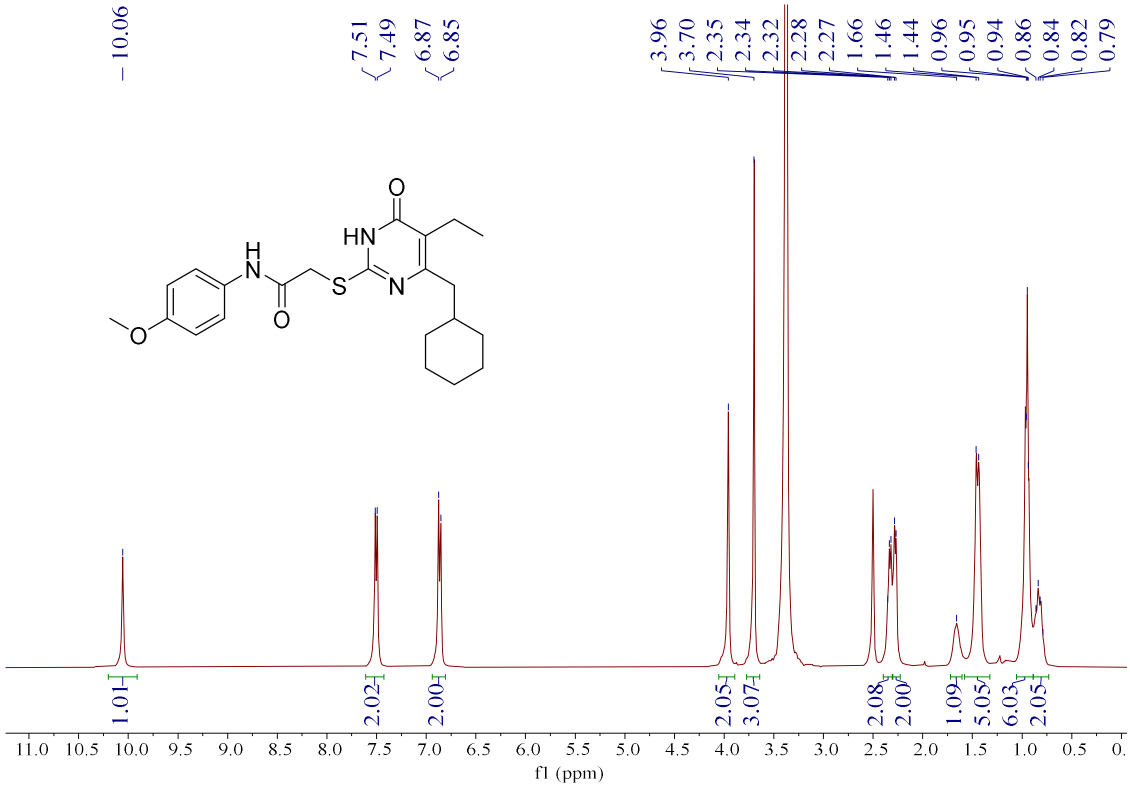
10.06
7.51
7.49
6.87
6.85
3.96
3.70
2.35
2.34
2.32
2.28
2.27
1.66
1.46
1.44
0.96
0.95
0.94
0.86
0.84
0.82
0.79
HN
O
N
H
N
S
O
O
1.01
2.02
2.00
2.05
3.07
2.08
2.00
1.09
5.05
6.03
2.05
11.0 10.5 10.0 9.5 9.0 8.5 8.0 7.5 7.0 6.5 6.0 5.5 5.0 4.5 4.0 3.5 3.0 2.5 2.0 1.5 1.0 0.5 0.
f1 (ppm)

4g

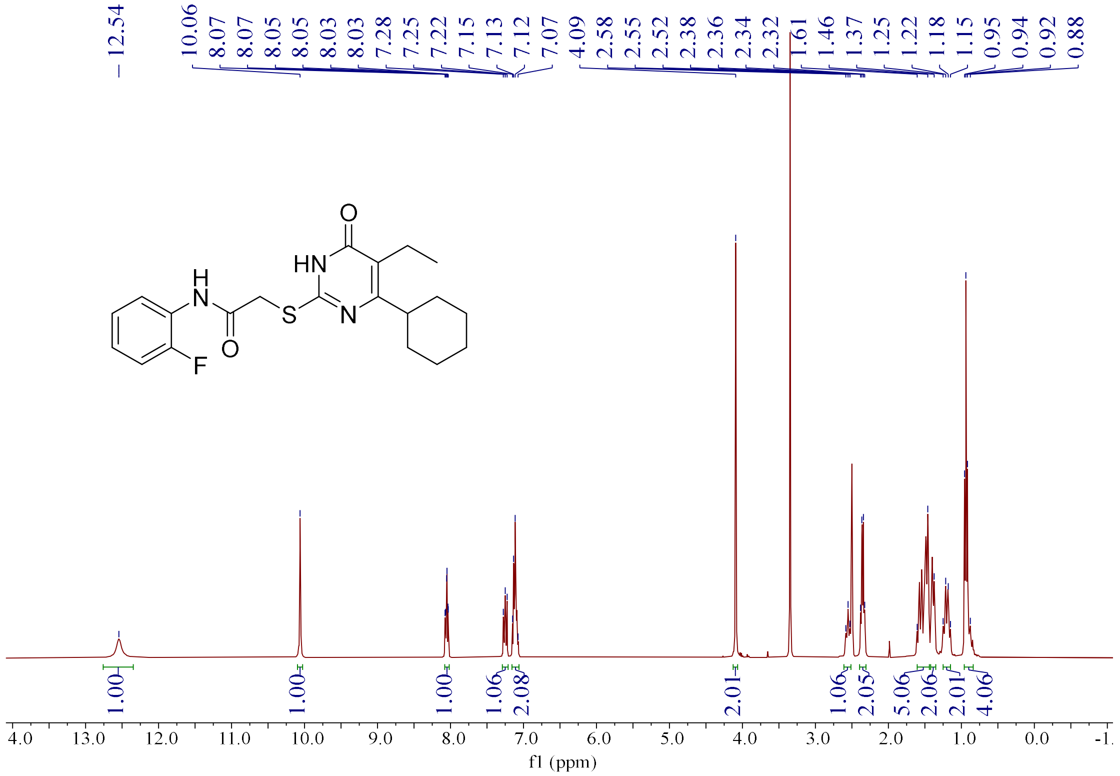
12.54
10.06
8.07
8.07
8.05
8.05
8.03
8.03
7.28
7.25
7.22
7.15
7.13
7.12
7.07
4.09
2.58
2.55
2.52
2.38
2.36
2.34
2.32
1.61
1.46
1.37
1.25
1.22
1.18
1.15
0.95
0.94
0.92
0.88
HN
O
N
H
N
S
O
F
1.00
1.00
1.00
1.06
2.08
2.01
1.06
2.05
5.06
2.06
2.01
4.06
4.0 13.0 12.0 11.0 10.0 9.0 8.0 7.0 6.0 5.0 4.0 3.0 2.0 1.0 0.0 -1.
f1 (ppm)

4h

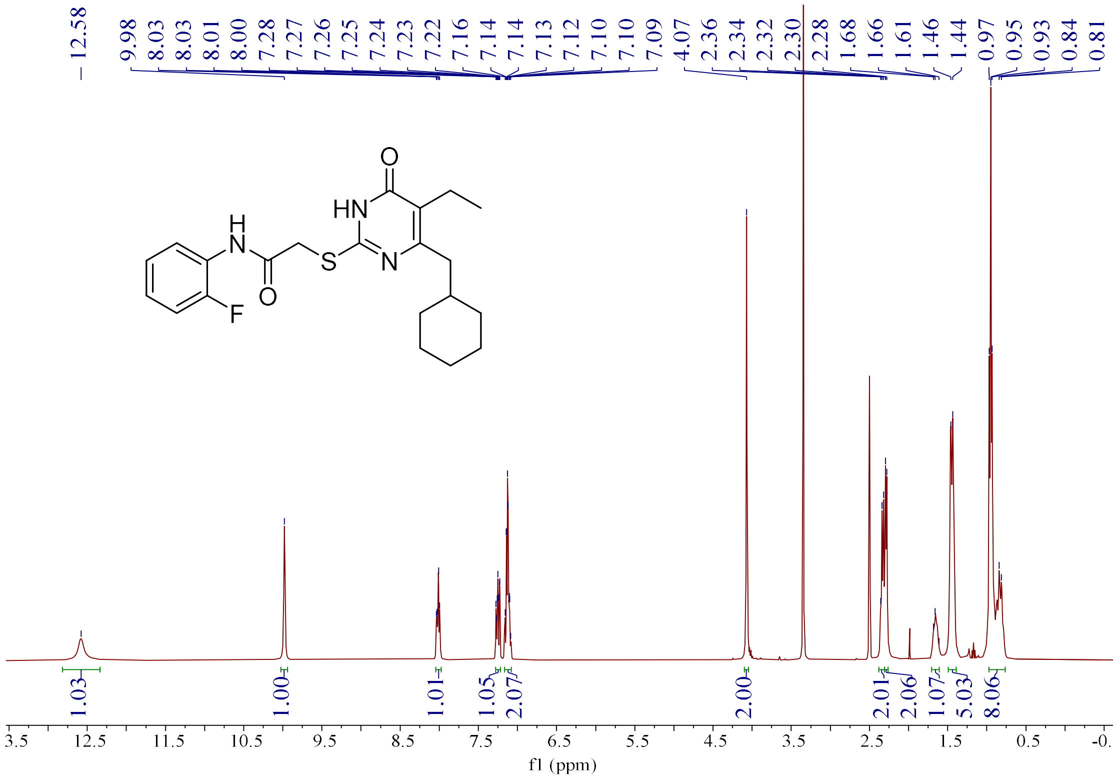

4i

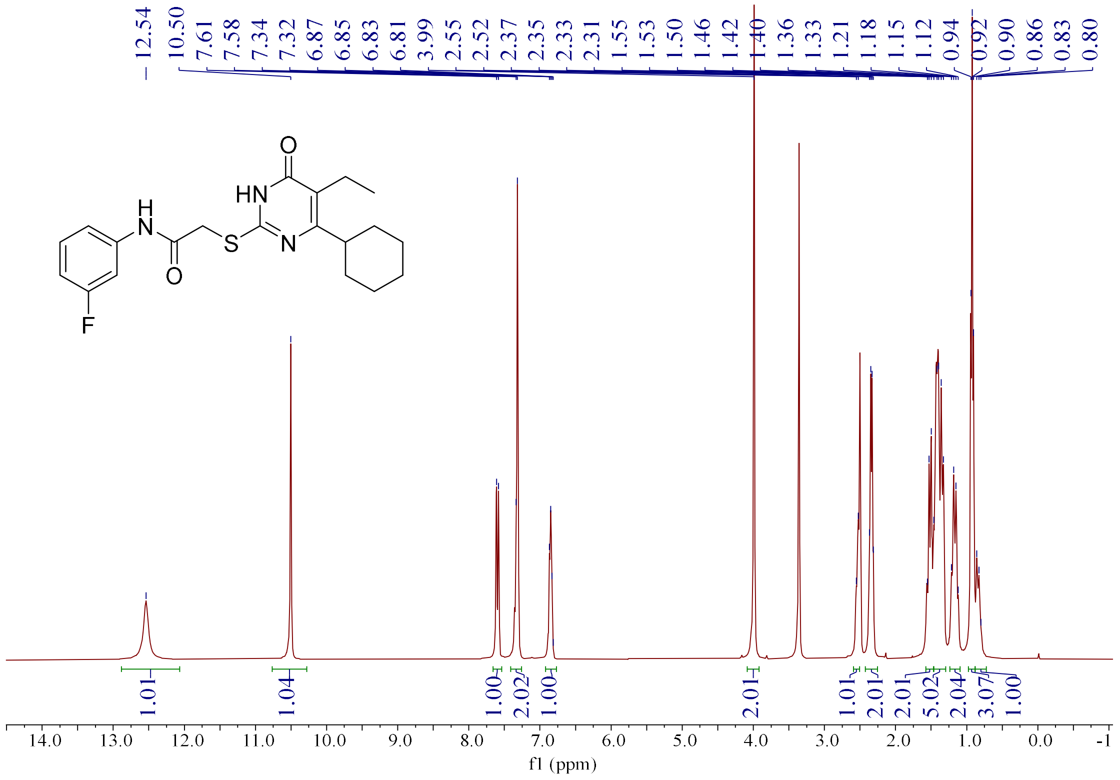

4j

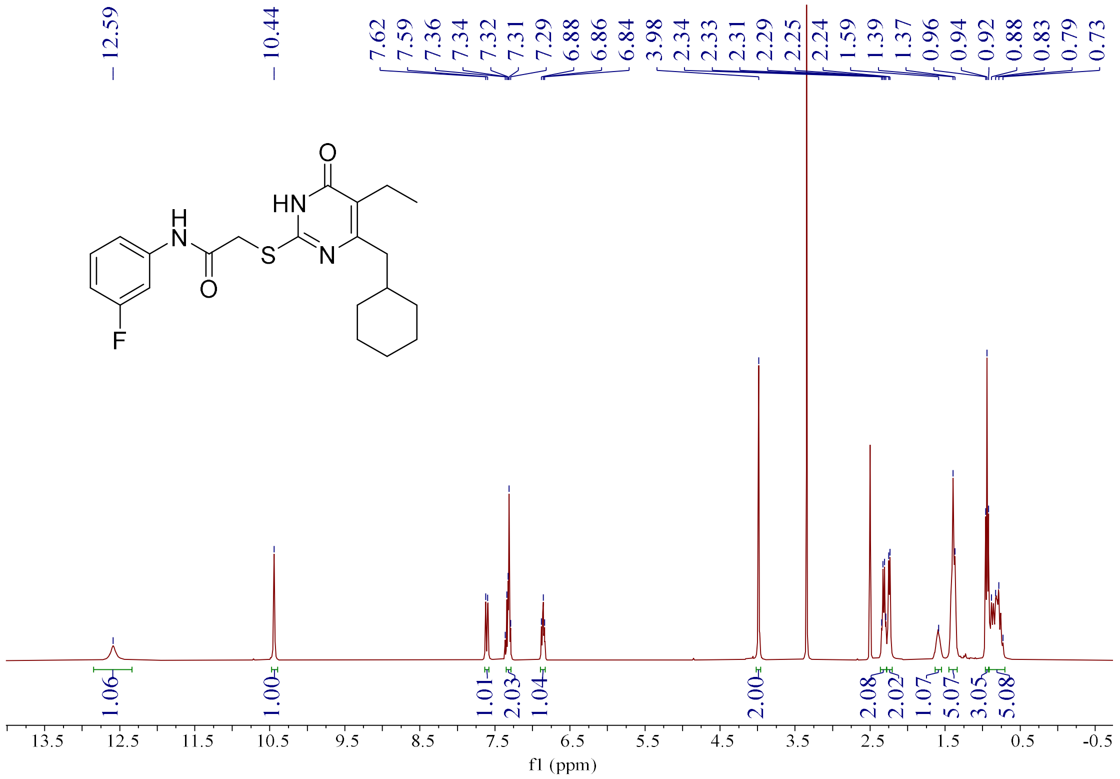

4k

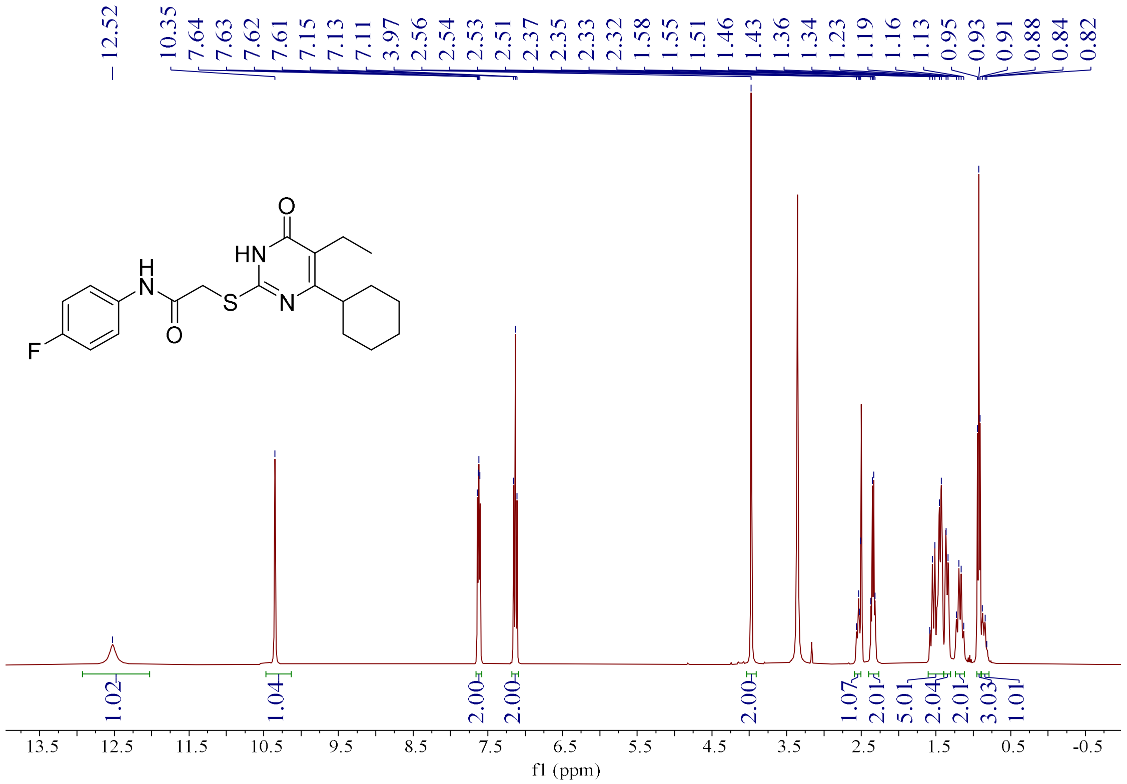

4l

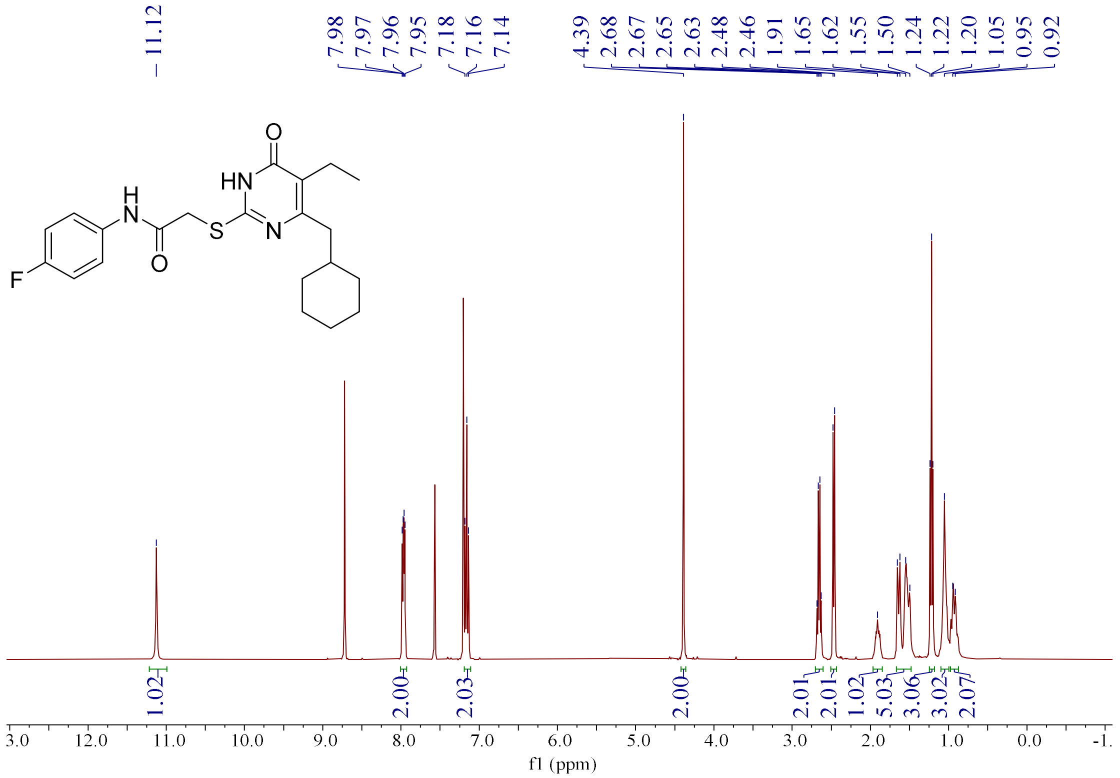

11.12
7.98
7.97
7.96
7.95
7.18
7.16
7.14
4.39
2.68
2.67
2.65
2.63
2.48
2.46
1.91
1.65
1.62
1.55
1.50
1.24
1.22
1.20
1.05
0.95
0.92
1.02
2.00
2.03
2.00
2.01
2.01
1.02
5.03
3.06
3.02
2.07
f1 (ppm)

4m

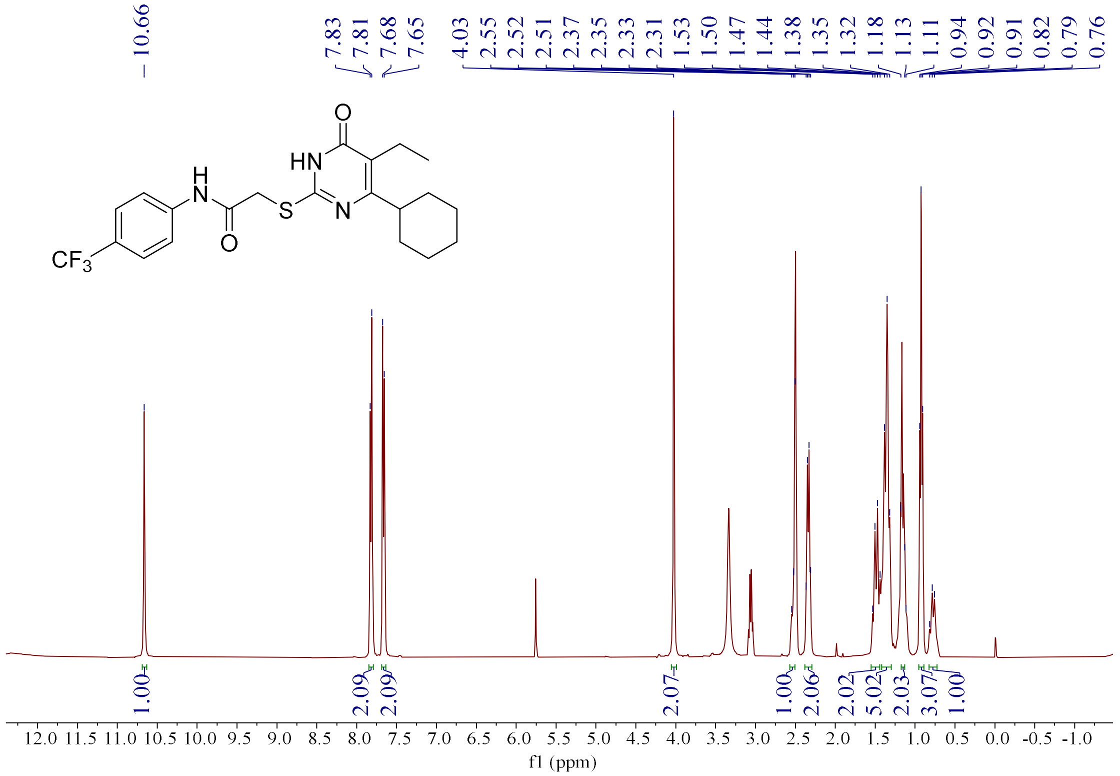

10.66
7.83
7.81
7.68
7.65
4.03
2.55
2.52
2.51
2.37
2.35
2.33
2.31
1.53
1.50
1.47
1.44
1.38
1.35
1.32
1.18
1.13
1.11
0.94
0.92
0.91
0.82
0.79
0.76
1.00
2.09
2.09
2.07
1.00
2.06
2.02
5.02
2.03
3.07
1.00
CF3
f1 (ppm)

4n

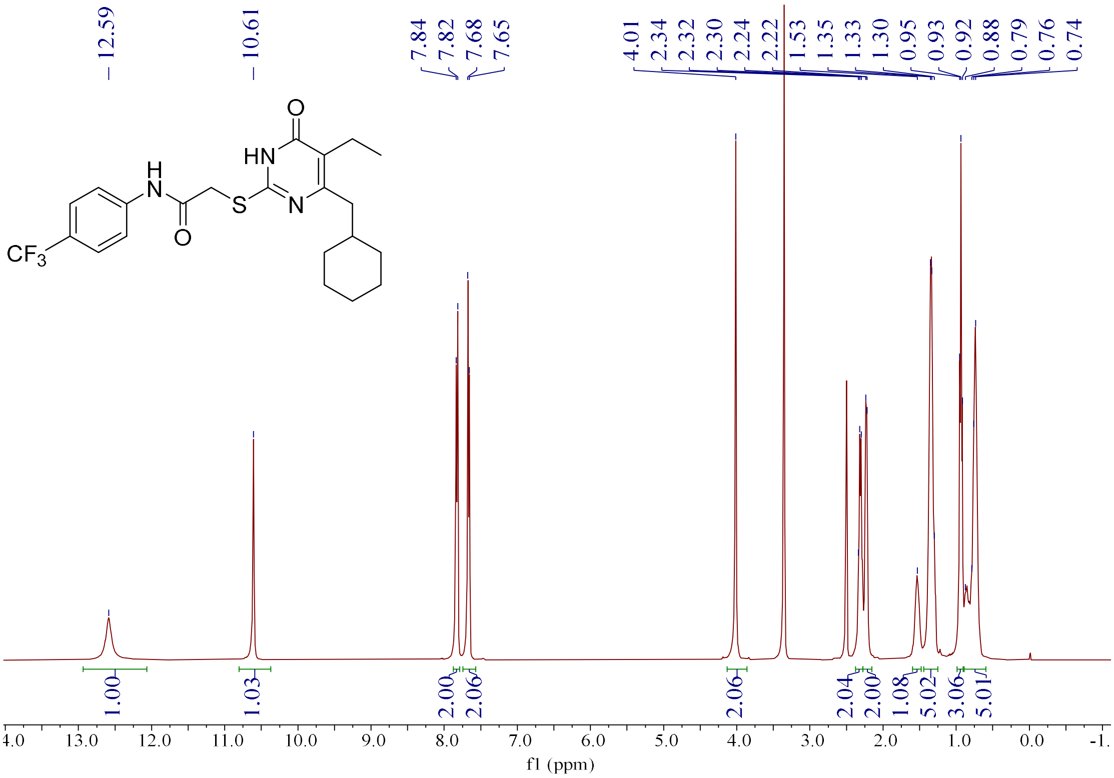
12.59
10.61
7.84
7.82
7.68
7.65
4.01
2.34
2.32
2.30
2.24
2.22
1.53
1.35
1.33
1.30
0.95
0.93
0.92
0.88
0.79
0.76
0.74
CF3
f1 (ppm)

4o

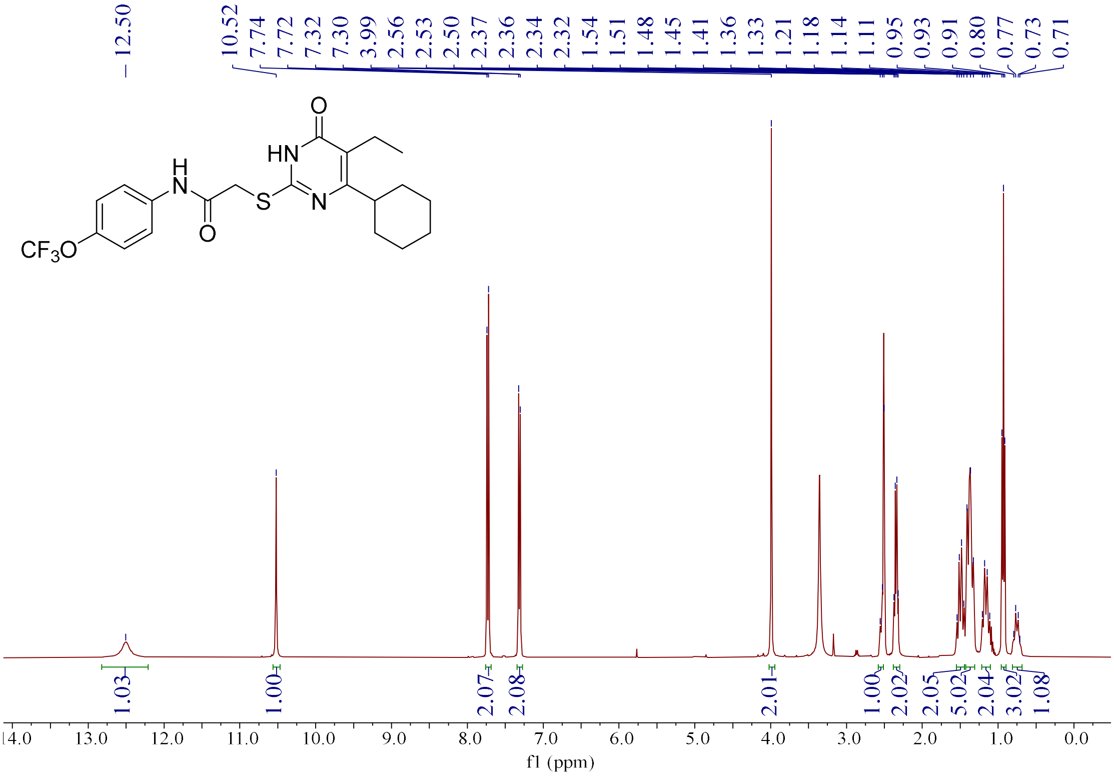
12.50
10.52
7.74
7.72
7.32
7.30
3.99
2.56
2.53
2.50
2.37
2.36
2.34
2.32
1.54
1.51
1.48
1.45
1.41
1.36
1.33
1.21
1.18
1.14
1.11
0.95
0.93
0.91
0.80
0.77
0.73
0.71
CF3O
f1 (ppm)

4p

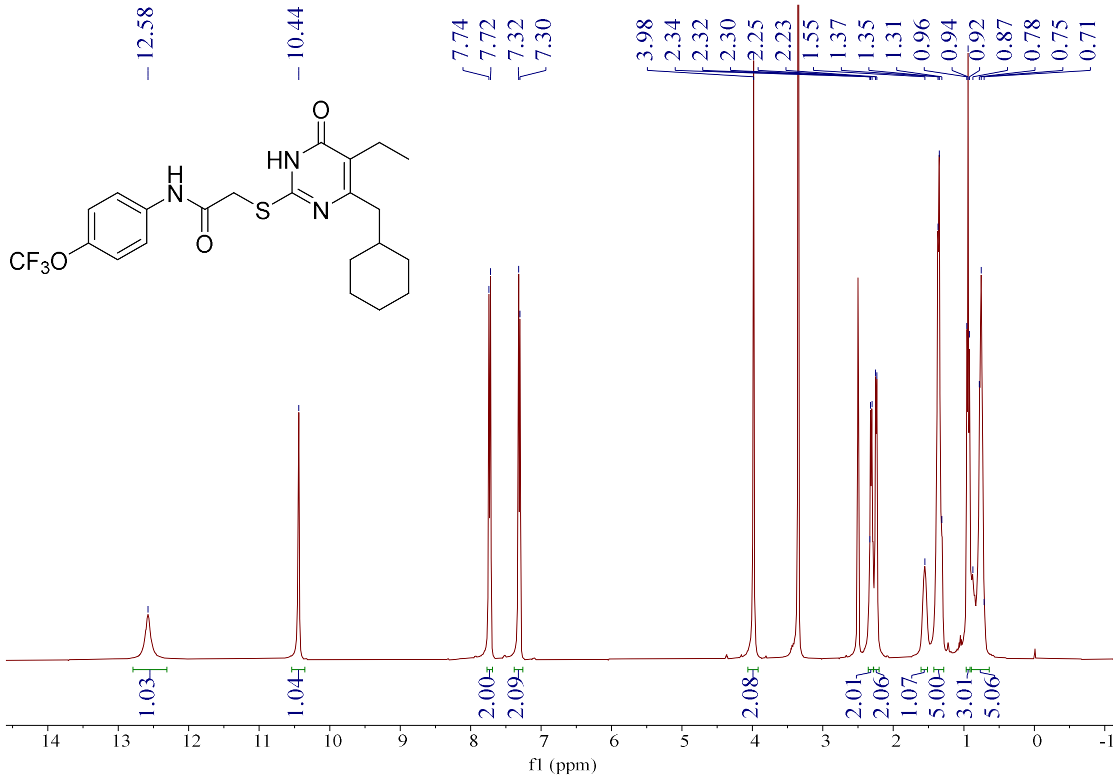
12.58
10.44
7.74
7.72
7.32
7.30
3.98
2.34
2.32
2.30
2.25
2.23
1.55
1.37
1.35
1.31
0.96
0.94
0.92
0.87
0.78
0.75
0.71
O
HN
H
N
S
N
O
CF3O
1.03
1.04
2.00
2.09
2.08
2.01
2.06
1.07
5.00
3.01
5.06
14
13
12
11
10
9
8
7
6
5
4
3
2
1
0
-1
f1 (ppm)

4q

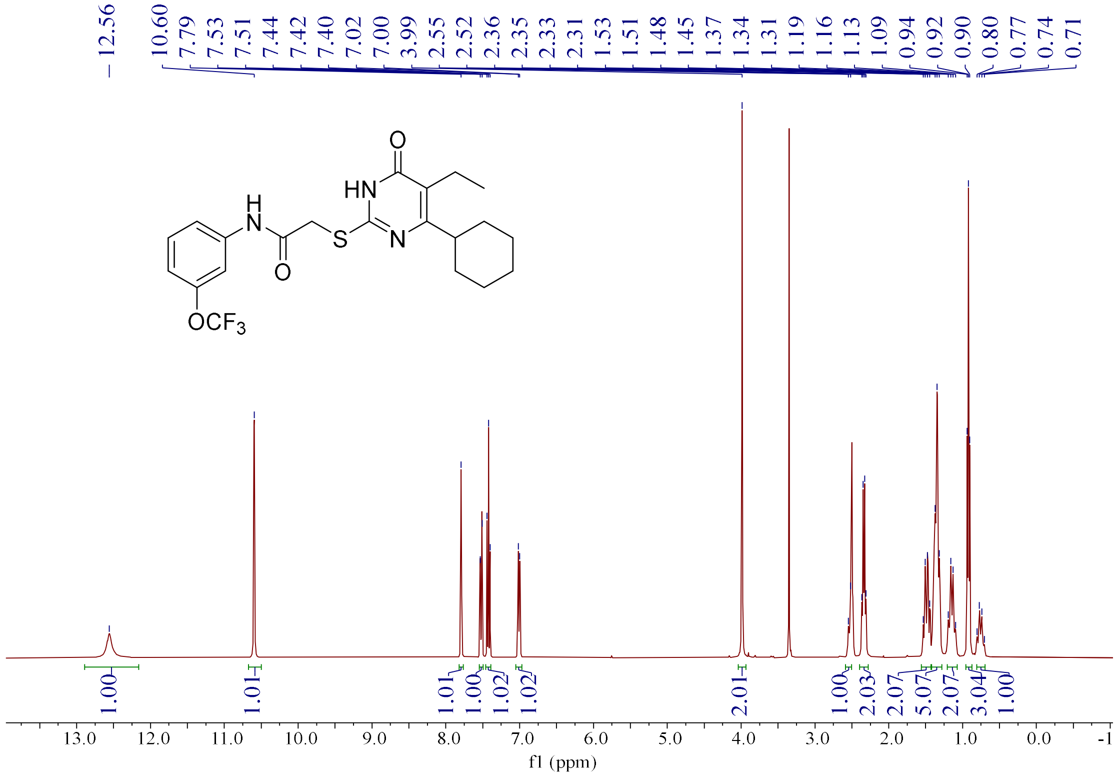
12.56
10.60
7.79
7.53
7.51
7.44
7.42
7.40
7.02
7.00
3.99
2.55
2.52
2.36
2.35
2.33
2.31
1.53
1.51
1.48
1.45
1.37
1.34
1.31
1.19
1.16
1.13
1.09
0.94
0.92
0.90
0.80
0.77
0.74
0.71
O
HN
H
N
S
N
O
OCF3
1.00
1.01
1.01
1.00
1.02
1.02
2.01
1.00
2.03
2.07
5.07
2.07
3.04
1.00
13.0
12.0
11.0
10.0
9.0
8.0
7.0
6.0
5.0
4.0
3.0
2.0
1.0
0.0
-1
f1 (ppm)

4r

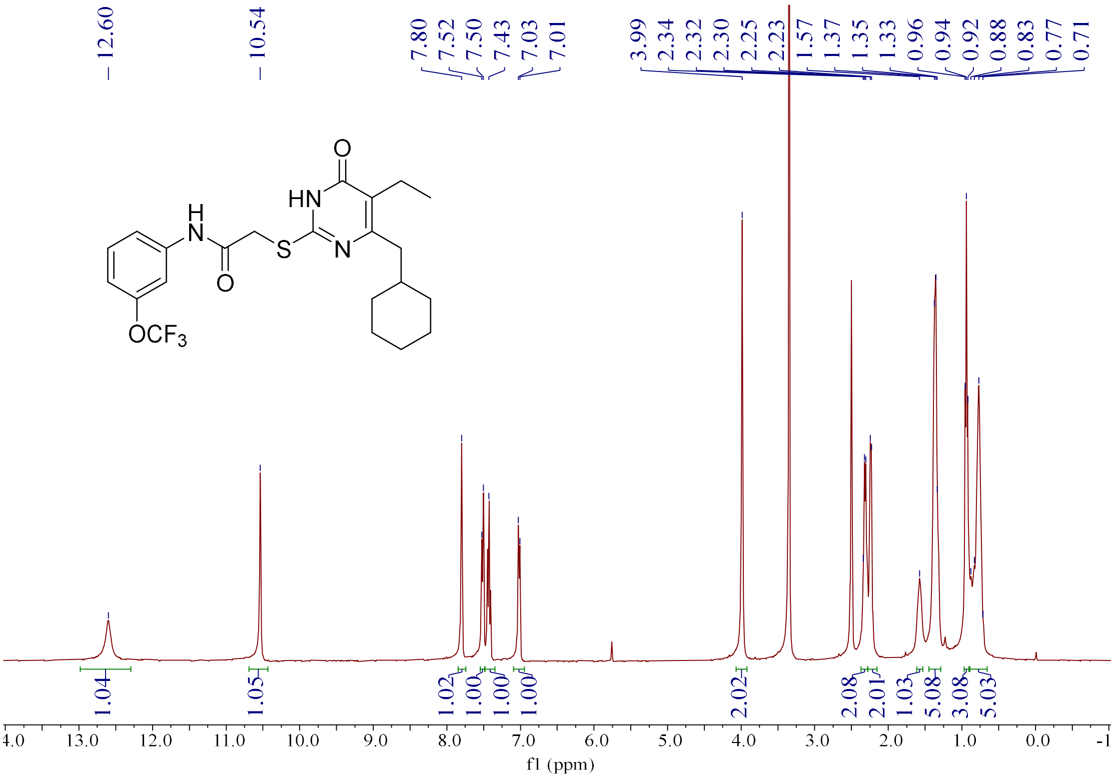
12.60
10.54
7.80 7.52 7.50 7.43 7.03 7.01
3.99 2.34 2.32 2.30 2.25 2.23 1.57 1.37 1.35 1.33 0.96 0.94 0.92 0.88 0.83 0.77 0.71
O
HN
H
N
S
N
O
OCF_3
1.04
1.05
1.02 1.00 1.00 1.00
2.02
2.08 2.01 1.03 5.08 3.08 5.03
f1 (ppm)

4s

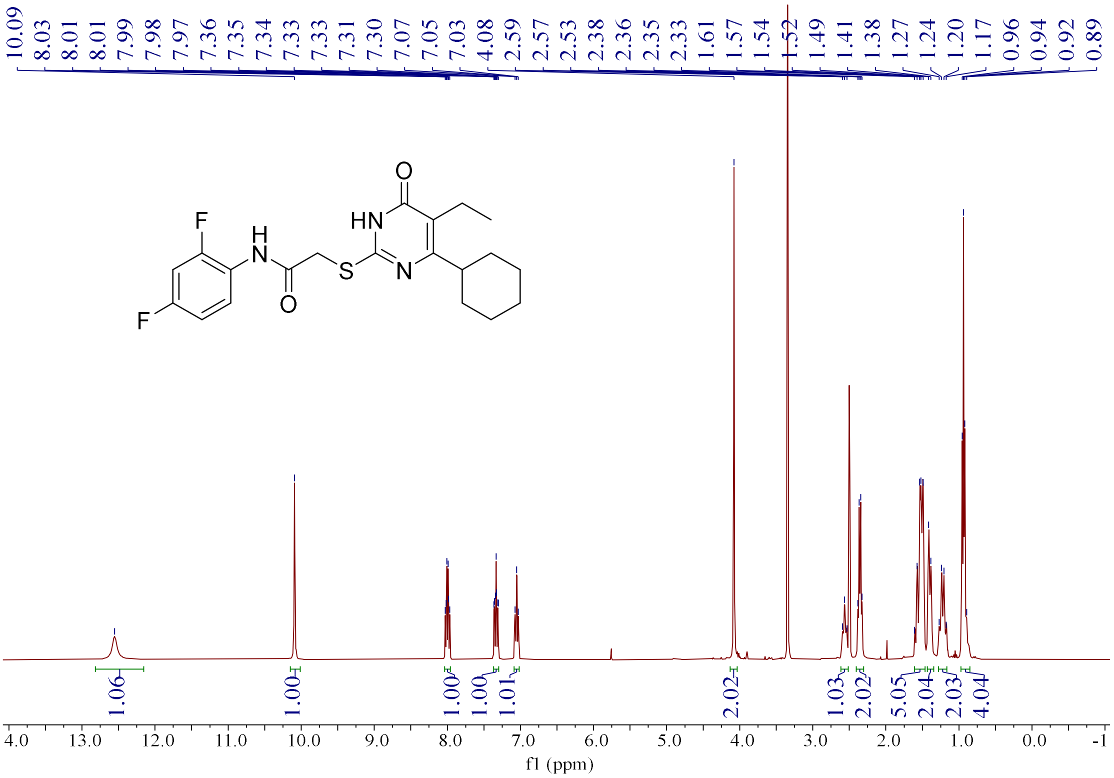
10.09 8.03 8.01 8.01 7.99 7.98 7.97 7.36 7.35 7.34 7.33 7.33 7.31 7.30 7.07 7.05 7.03 4.08 2.59 2.57 2.53 2.38 2.36 2.35 2.33 1.61 1.57 1.54 1.52 1.49 1.41 1.38 1.27 1.24 1.20 1.17 0.96 0.94 0.92 0.89
O
F
H
N
HN
S
N
O
F
1.06
1.00
1.00 1.00 1.01
2.02
1.03 2.02 5.05 2.04 2.03 4.04
f1 (ppm)

4t

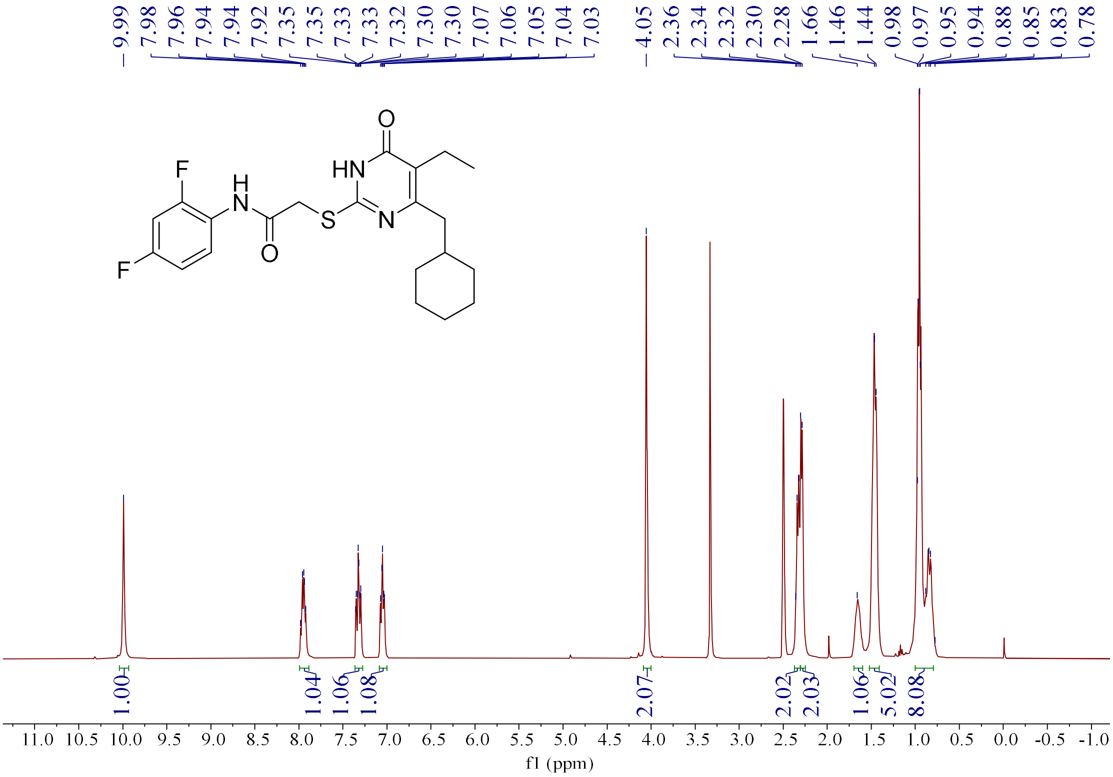
9.99 7.98 7.96 7.94 7.94 7.92 7.35 7.35 7.33 7.33 7.32 7.30 7.30 7.07 7.06 7.05 7.04 7.03 4.05 2.36 2.34 2.32 2.30 2.28 1.66 1.46 1.44 0.98 0.97 0.95 0.94 0.88 0.85 0.83 0.78
1.00 1.04 1.06 1.08 2.07 2.02 2.03 1.06 5.02 8.08
f1 (ppm)

4u

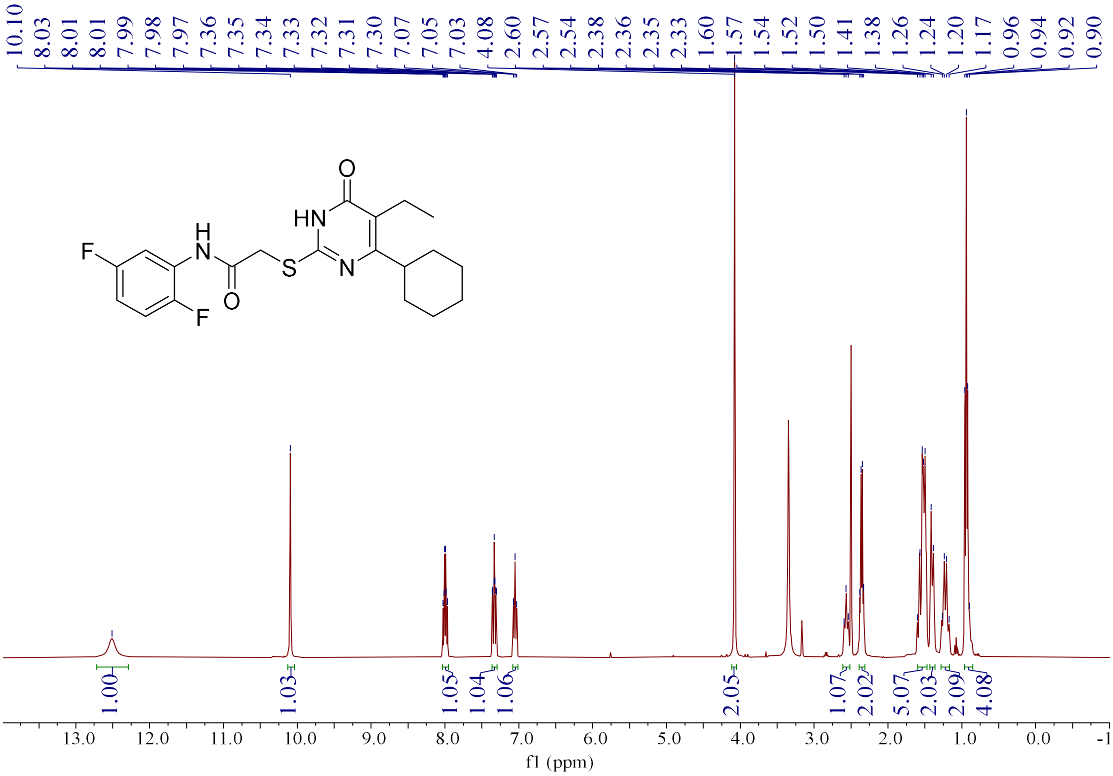
10.10 8.03 8.01 8.01 7.99 7.98 7.97 7.36 7.35 7.34 7.33 7.32 7.31 7.30 7.07 7.05 7.03 4.08 2.60 2.57 2.54 2.38 2.36 2.35 2.33 1.60 1.57 1.54 1.52 1.50 1.41 1.38 1.26 1.24 1.20 1.17 0.96 0.94 0.92 0.90
1.00 1.03 1.05 1.04 1.06 2.05 1.07 2.02 5.07 2.03 2.09 4.08
f1 (ppm)

4v

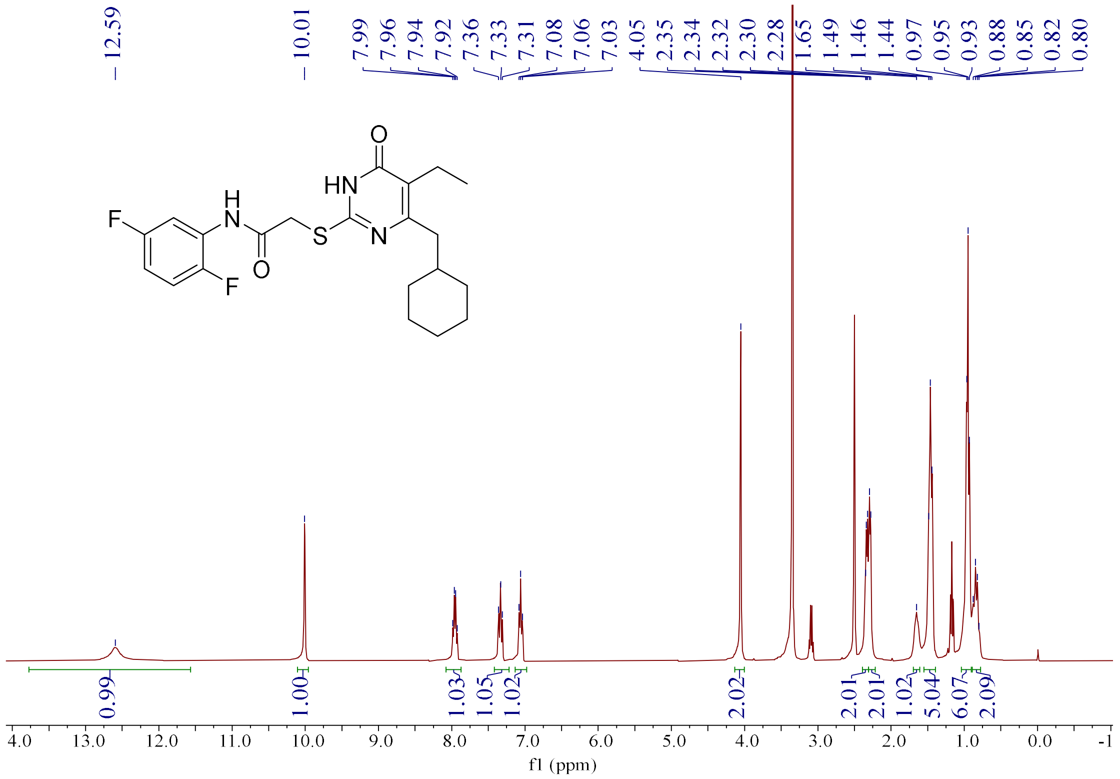

4w

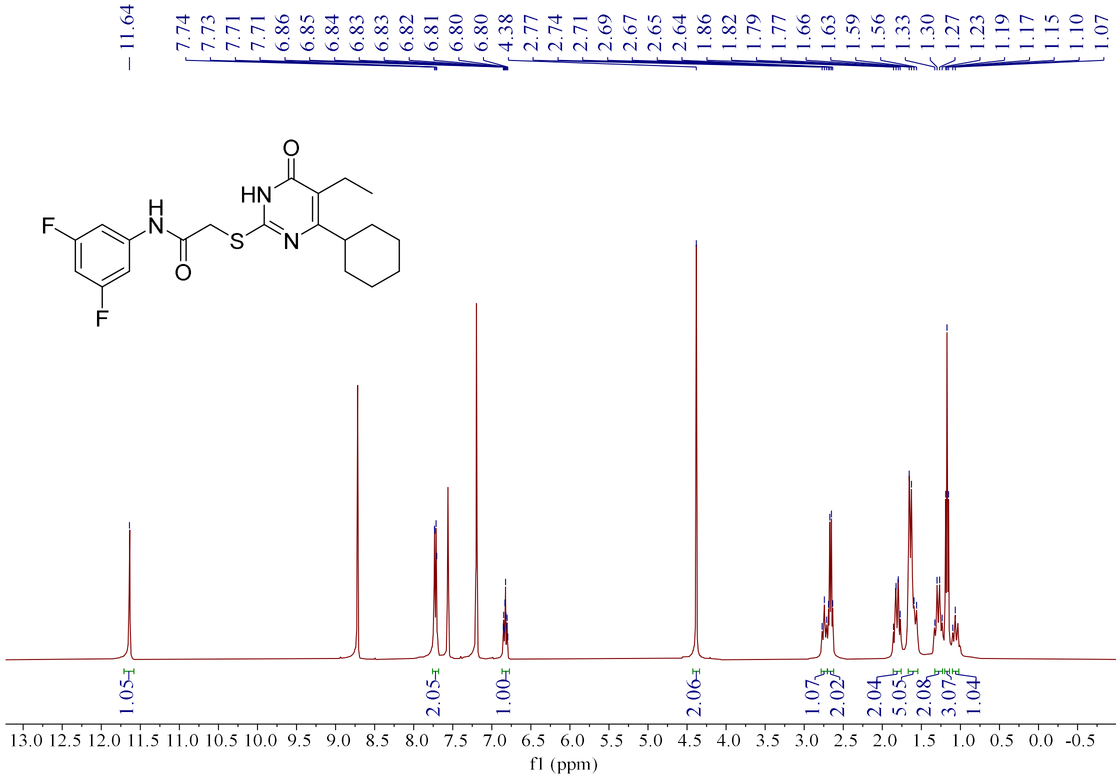

4x

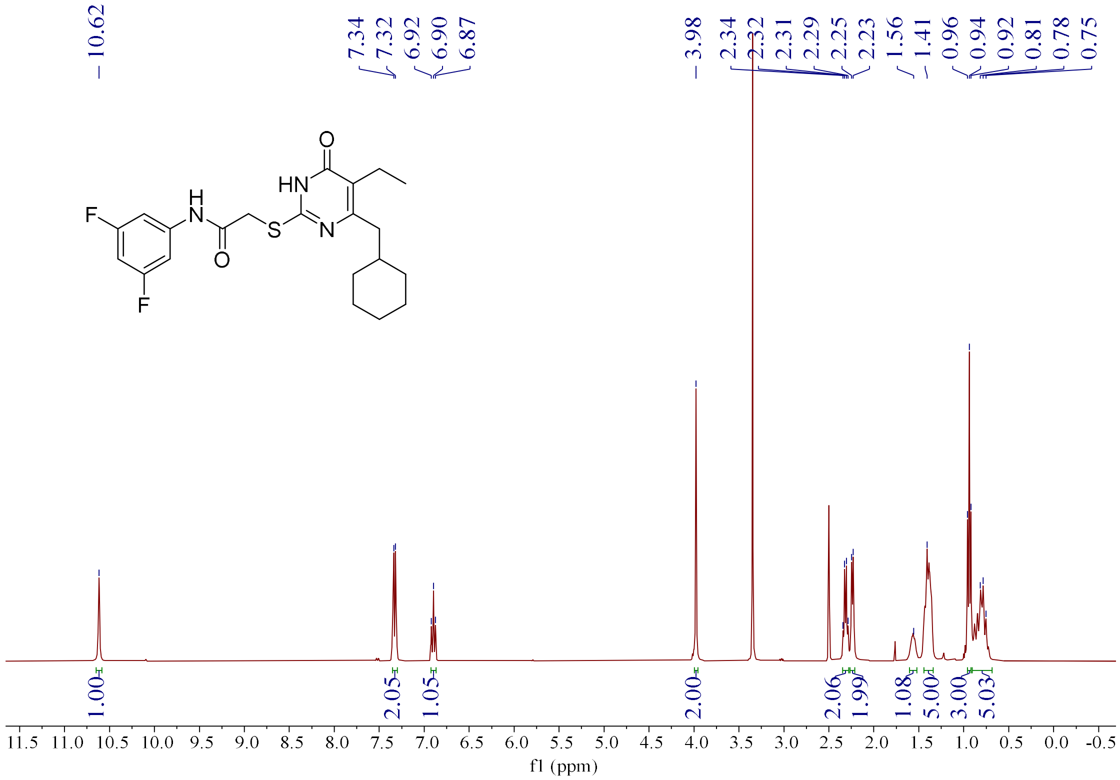
10.62
7.34
7.32
6.92
6.90
6.87
3.98
2.34
2.32
2.31
2.29
2.25
2.23
1.56
1.41
0.96
0.94
0.92
0.81
0.78
0.75
F
H
N
O
S
HN
N
O
F
1.00
2.05
1.05
2.00
2.06
1.99
1.08
5.00
3.00
5.03
11.5 11.0 10.5 10.0 9.5 9.0 8.5 8.0 7.5 7.0 6.5 6.0 5.5 5.0 4.5 4.0 3.5 3.0 2.5 2.0 1.5 1.0 0.5 0.0 -0.5
f1 (ppm)

4y

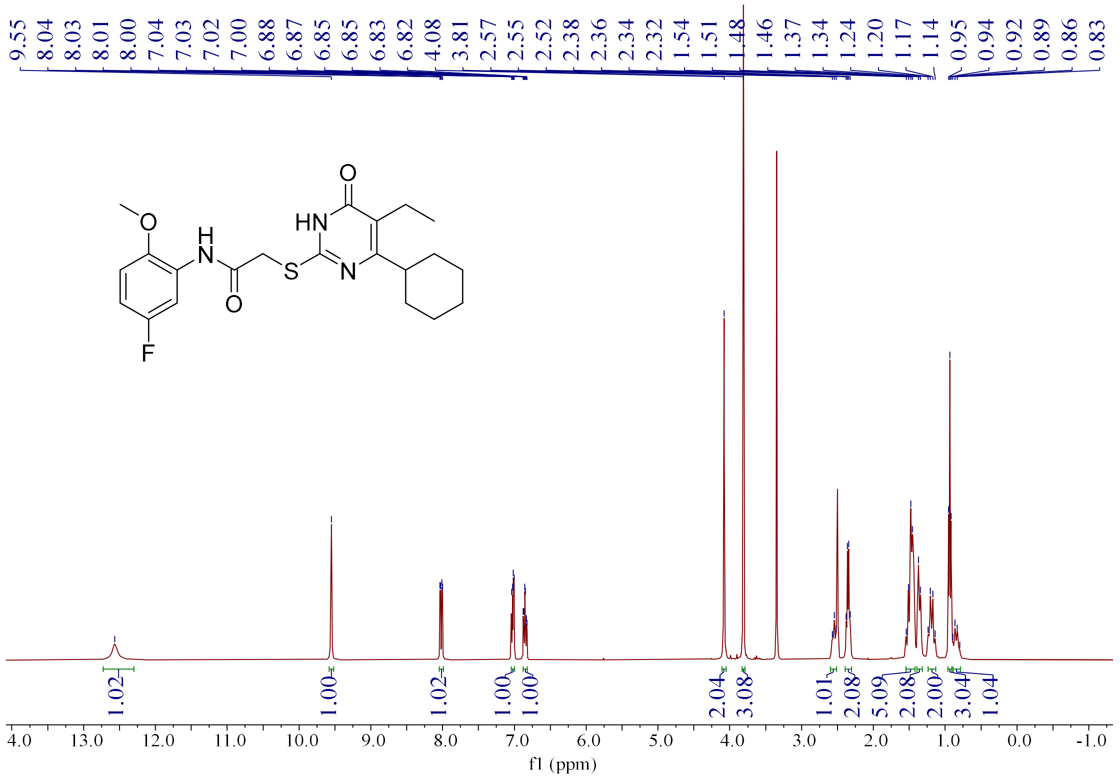
9.55
8.04
8.03
8.01
8.00
7.04
7.03
7.02
7.00
6.88
6.87
6.85
6.85
6.83
6.82
4.08
3.81
2.57
2.55
2.52
2.38
2.36
2.34
2.32
1.54
1.51
1.48
1.46
1.37
1.34
1.24
1.20
1.17
1.14
0.95
0.94
0.92
0.89
0.86
0.83
O
H
N
O
S
HN
N
O
F
1.02
1.00
1.02
1.00
1.00
2.04
3.08
1.01
2.08
5.09
2.08
2.00
3.04
1.04
4.0 13.0 12.0 11.0 10.0 9.0 8.0 7.0 6.0 5.0 4.0 3.0 2.0 1.0 0.0 -1.0
f1 (ppm)

4z

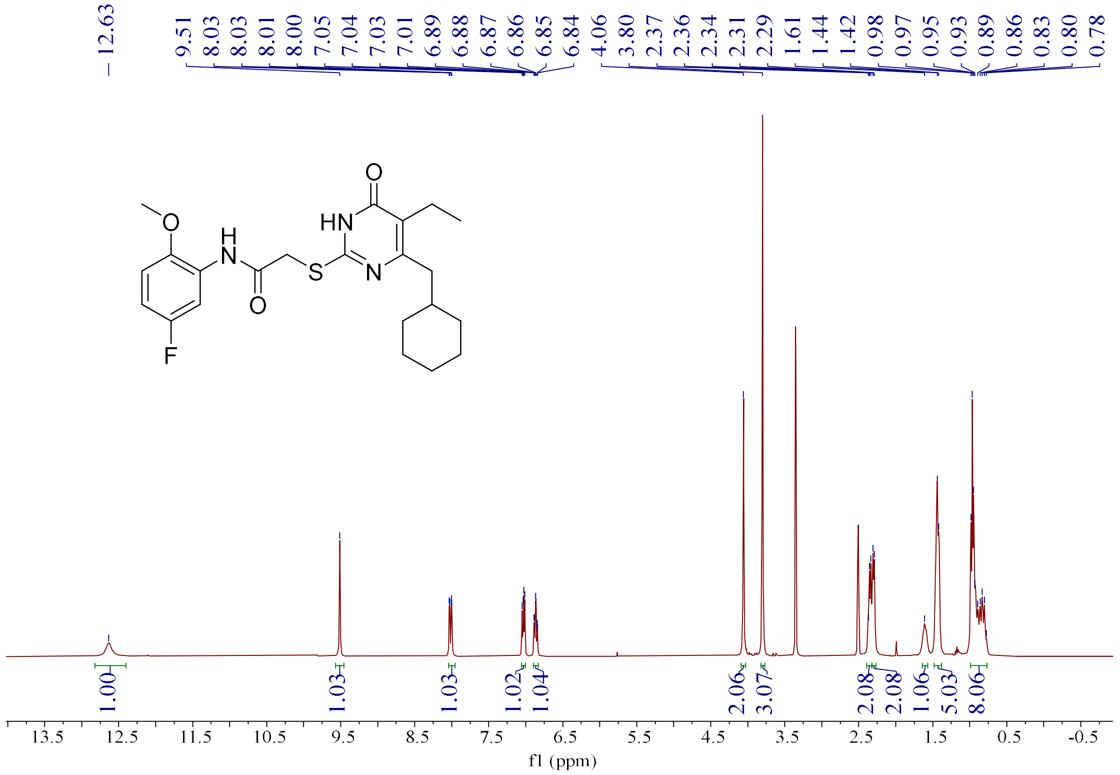

ESI MS

4g

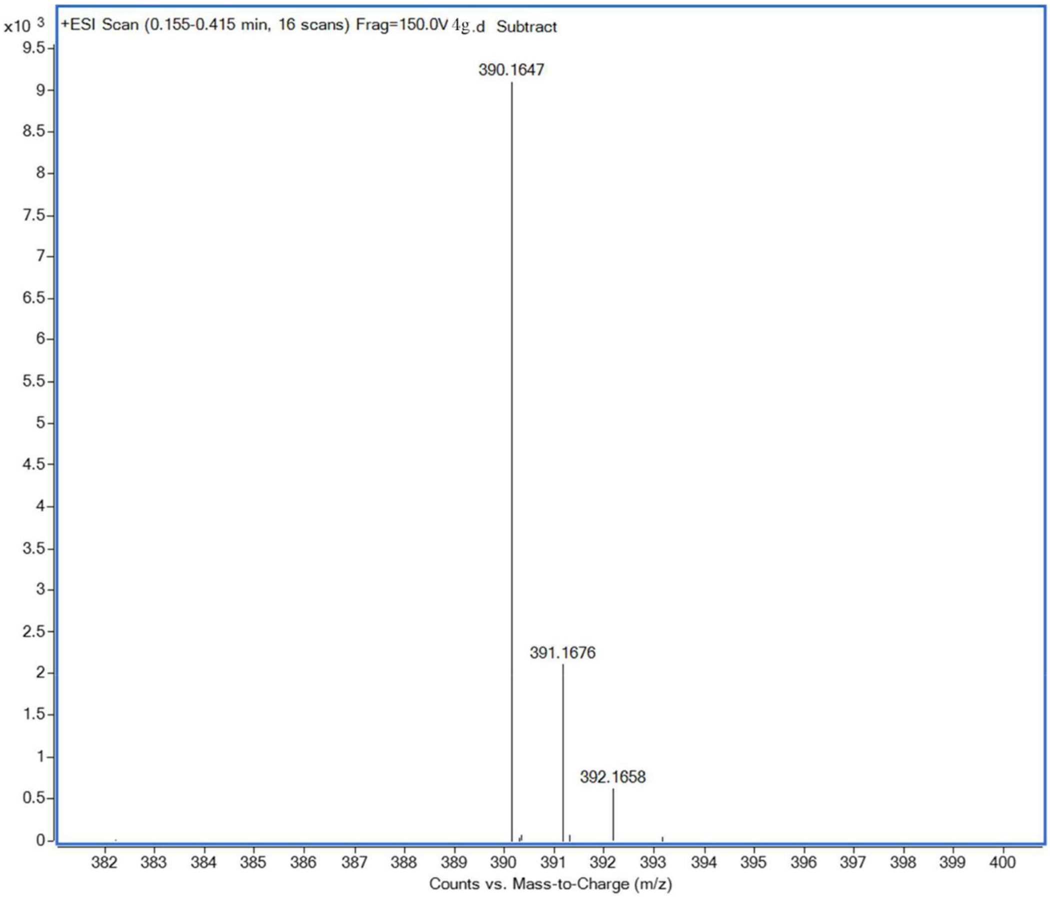

MS Formula Results: + Scan (0.155-0.415 min) Sub (4g.d)

m/z	Ion	Formula	Abundance
390.1647	(M+H)+	C20 H25 F N3 O2 S	9099

Best	Formula (M)	Ion Formula	Score	Cross Score	Calc m/z	Diff (ppm)	Mass Match	Abund Match	Spacing Match
✓	C20 H24 F N3 O2 S	C20 H25 F N3 O2 S	99.39		390.1646	-0.21	99.96	99.29	98.37

4h

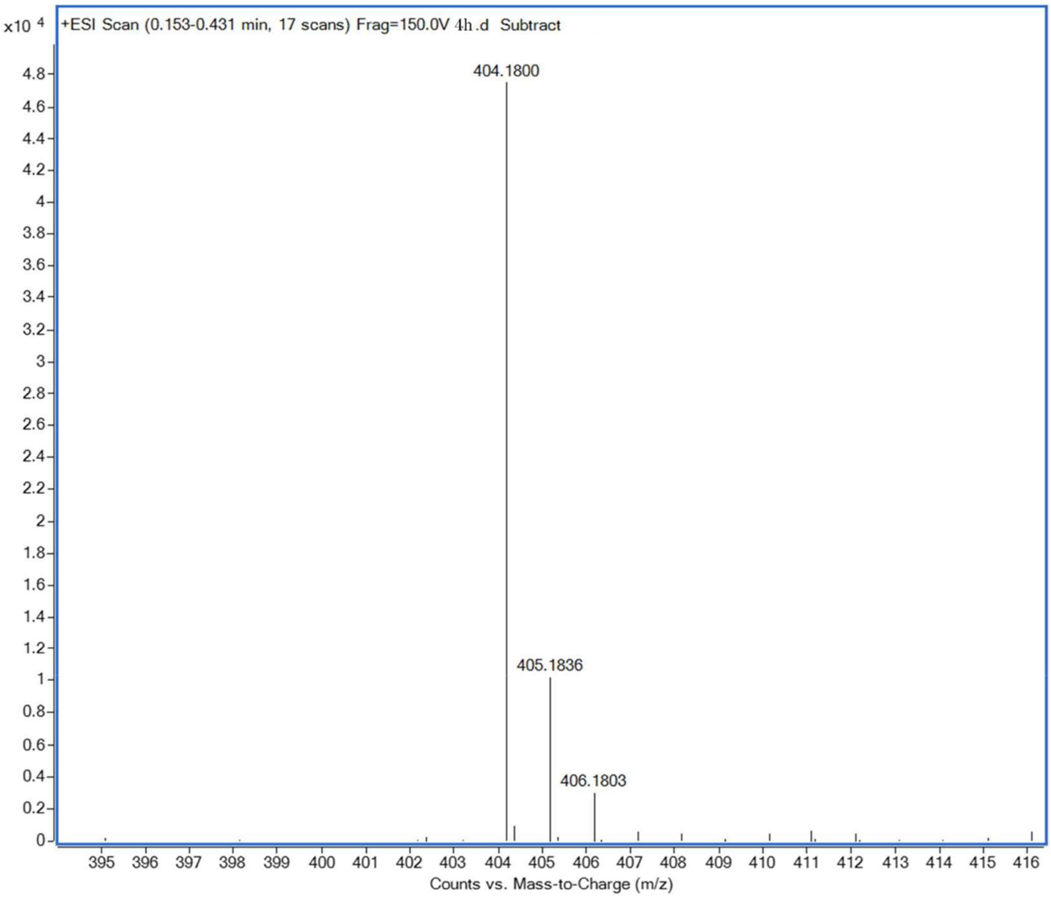

MS Formula Results: + Scan (0.153-0.431 min) Sub (4h .d)

m/z	Ion	Formula	Abundance
404.18	(M+H)+	C21 H27 F N3 O2 S	47555.1

Best	Formula (M)	Ion Formula	Score	Cross Score	Calc m/z	Diff (ppm)	Mass Match	Abund Match	Spacing Match
✔	C21 H26 F N3 O2 S	C21 H27 F N3 O2 S	97.09		404.1803	0.68	99.59	91.93	98.3

4i

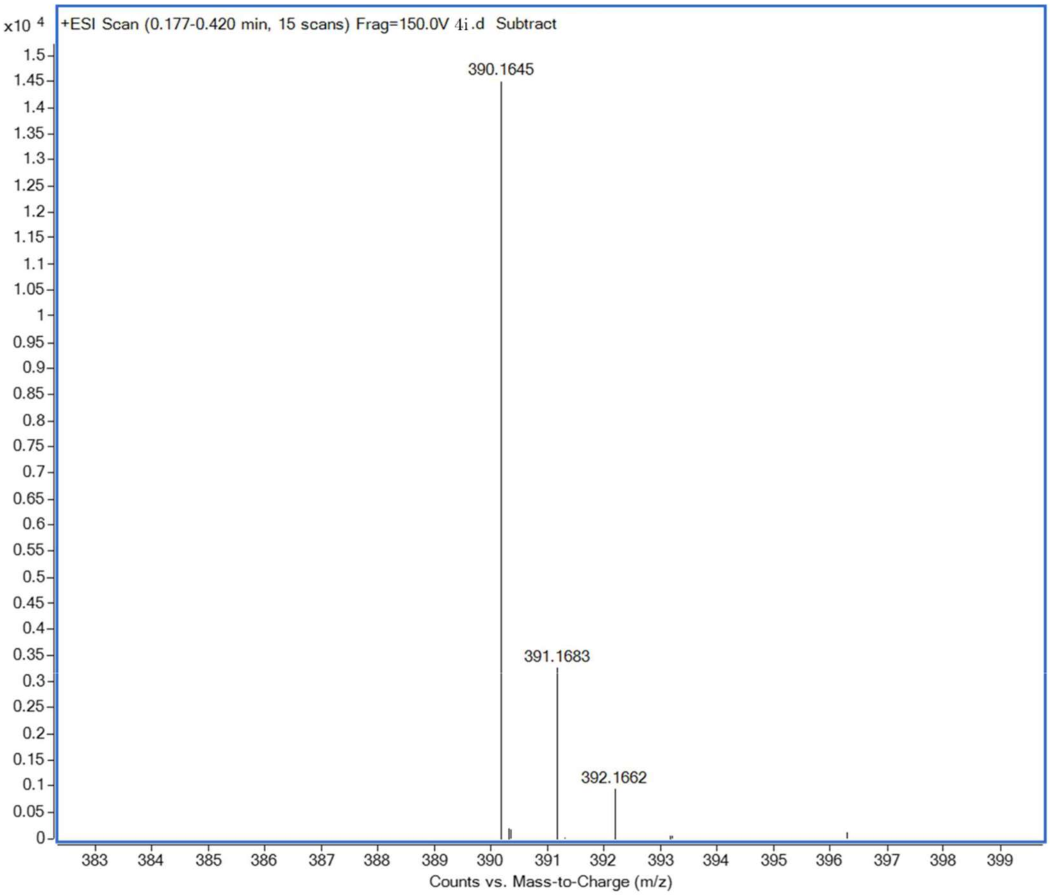

MS Formula Results: + Scan (0.177-0.420 min) Sub (4i .d)

m/z	Ion	Formula	Abundance
390.1645	(M+H)+	C20 H25 F N3 O2 S	14505.4

Best	Formula (M)	Ion Formula	Score	Cross Score	Calc m/z	Diff (ppm)	Mass Match	Abund Match	Spacing Match
✔	C20 H24 F N3 O2 S	C20 H25 F N3 O2 S	97.9		390.1646	0.23	99.95	97.91	93.81

4j

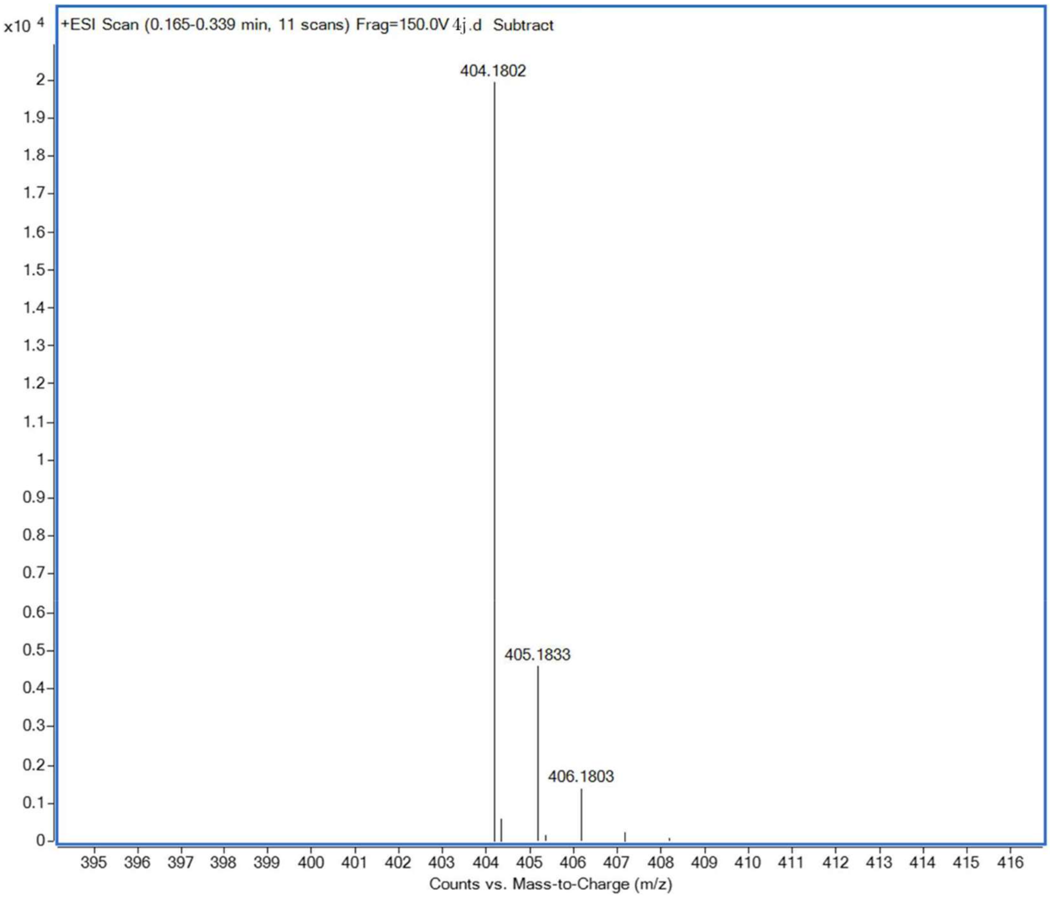

MS Formula Results: + Scan (0.165-0.339 min) Sub (4j .d)

m/z	Ion	Formula	Abundance
404.1802	(M+H)+	C21 H27 F N3 O2 S	19942.5

Best	Formula (M)	Ion Formula	Score	Cross Score	Calc m/z	Diff (ppm)	Mass Match	Abund Match	Spacing Match
✔	C21 H26 F N3 O2 S	C21 H27 F N3 O2 S	99.32		404.1803	0.22	99.96	97.84	99.82

4n

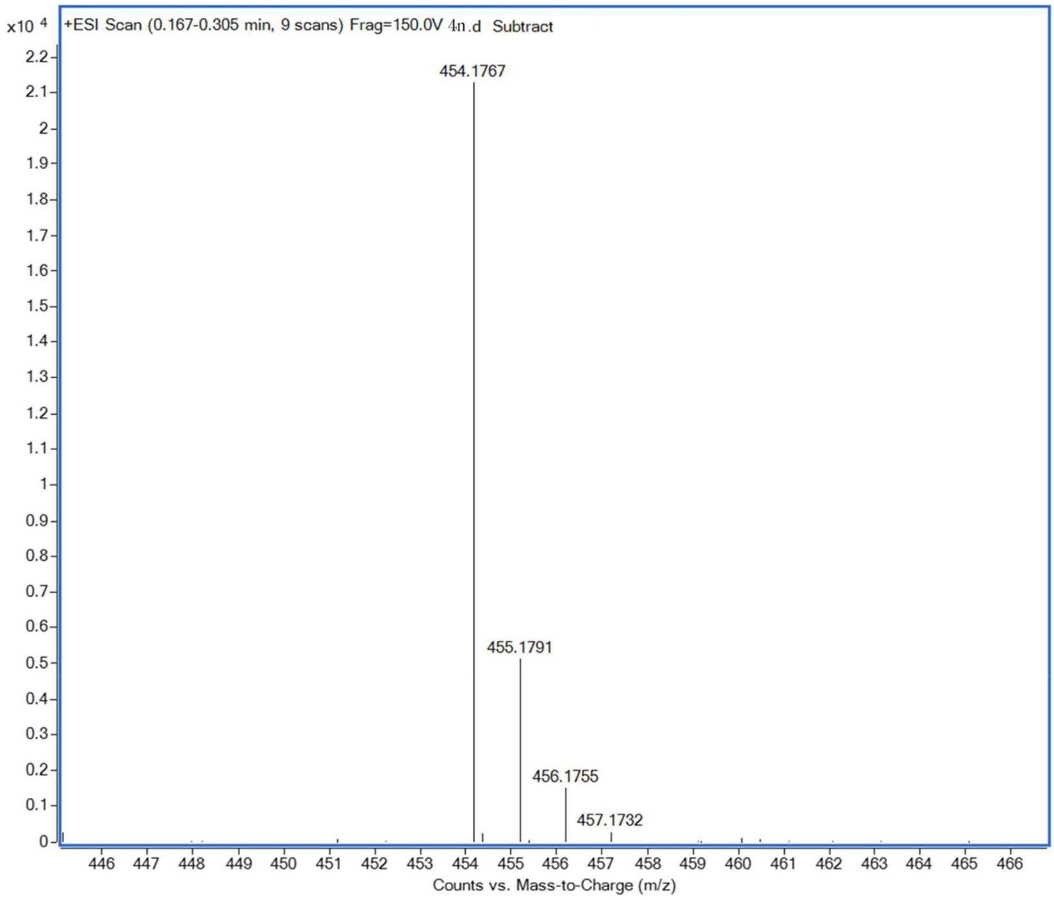

MS Formula Results: + Scan (0.167-0.305 min) Sub (4n.d)

m/z	Ion	Formula	Abundance
454.1767	(M+H)+	C22 H27 F3 N3 O2 S	21300.8

Best	Formula (M)	Ion Formula	Score	Cross Score	Calc m/z	Diff (ppm)	Mass Match	Abund Match	Spacing Match
✔	C22 H26 F3 N3 O2 S	C22 H27 F3 N3 O2 S	98.11		454.1771	0.85	99.29	97.57	96.39

4r

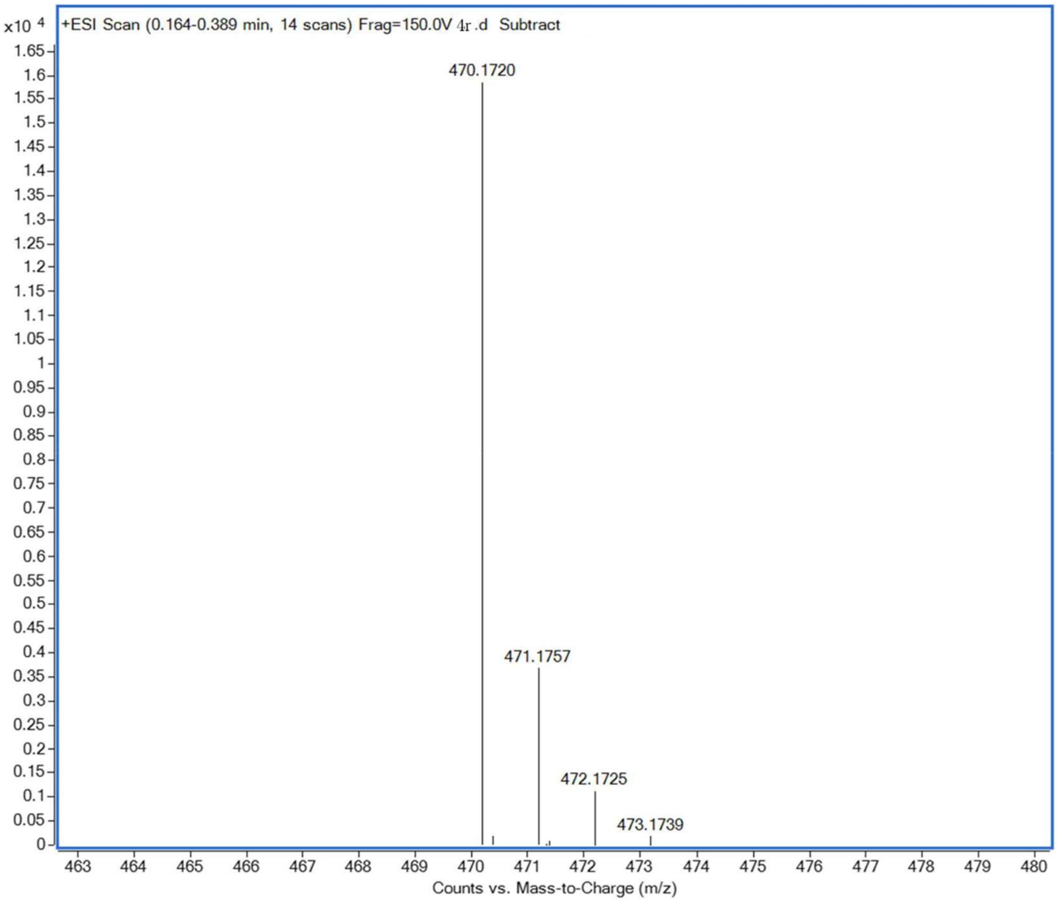

MS Formula Results: + Scan (0.164-0.389 min) Sub (4r.d)

m/z	Ion	Formula	Abundance
470.172	(M+H)+	C22 H27 F3 N3 O3 S	15856.9

Best	Formula (M)	Ion Formula	Score	Cross Score	Calc m/z	Diff (ppm)	Mass Match	Abund Match	Spacing Match
✔	C22 H26 F3 N3 O3 S	C22 H27 F3 N3 O3 S	98.19		470.172	-0.02	100	95.35	97.96

4t

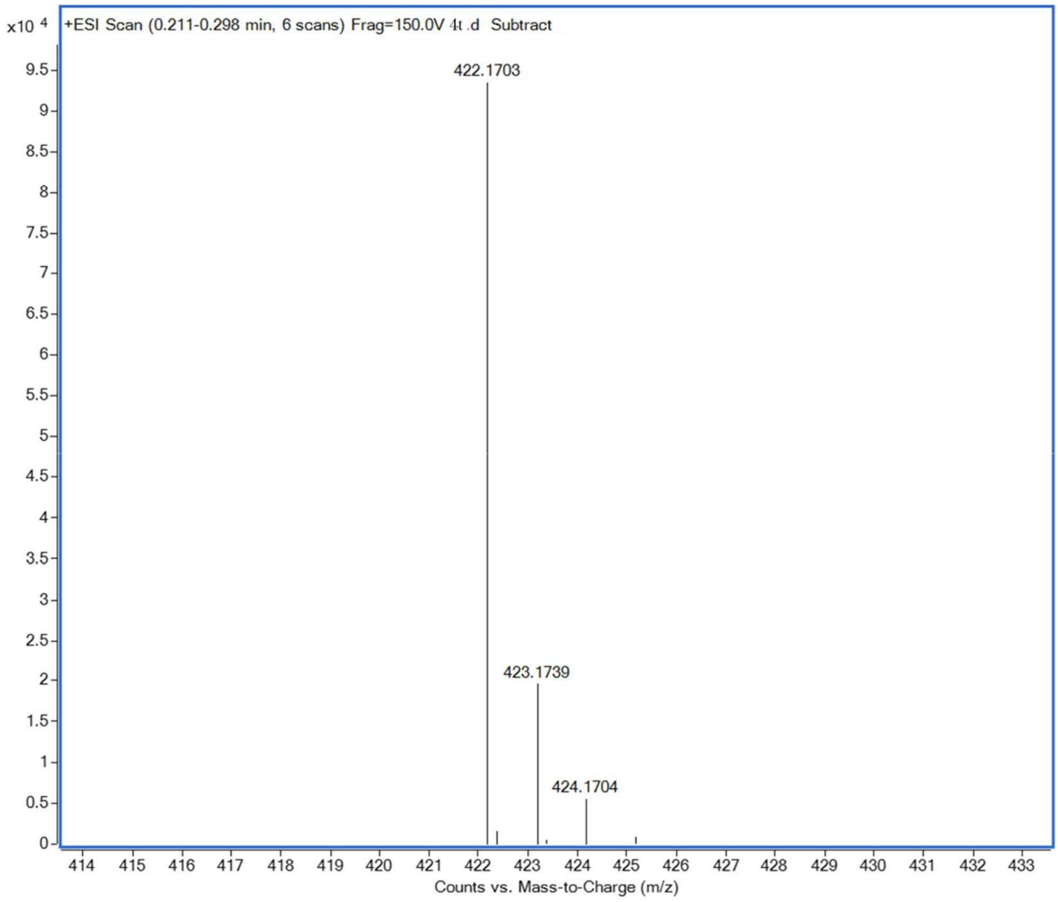

MS Formula Results: + Scan (0.211-0.298 min) Sub (4t .d)

m/z	Ion	Formula	Abundance
422.1703	(M+H)+	C21 H26 F2 N3 O2 S	93481.9

Best	Formula (M)	Ion Formula	Score	Cross Score	Calc m/z	Diff (ppm)	Mass Match	Abund Match	Spacing Match
✓	C21 H25 F2 N3 O2 S	C21 H26 F2 N3 O2 S	95.98		422.1708	1.34	98.35	90.17	98.23

4w

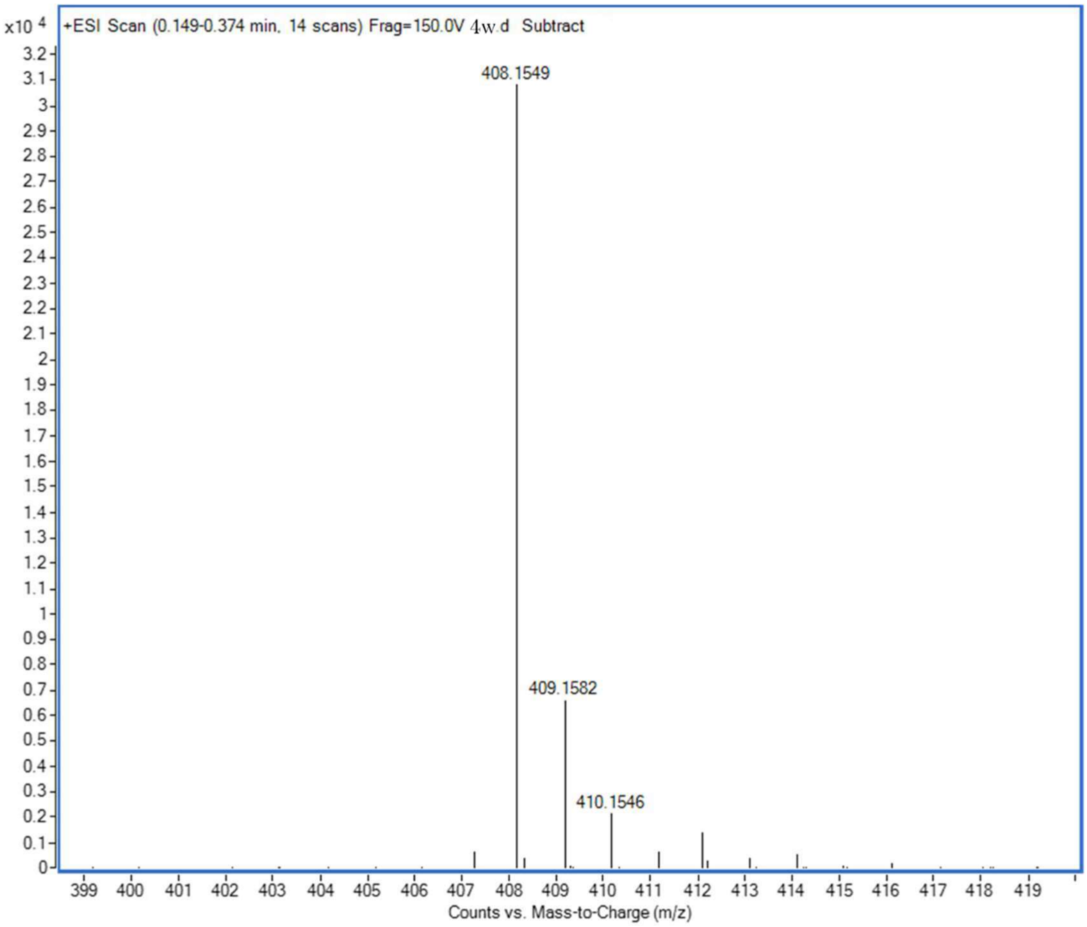

MS Formula Results: + Scan (0.149-0.374 min) Sub (4w.d)

m/z	Ion	Formula	Abundance
408.1549	(M+H)+	C20 H24 F2 N3 O2 S	30820.5

Best	Formula (M)	Ion Formula	Score	Cross Score	Calc m/z	Diff (ppm)	Mass Match	Abund Match	Spacing Match
✓	C20 H23 F2 N3 O2 S	C20 H24 F2 N3 O2 S	98.62		408.1552	0.59	99.69	96.14	99.44
